# STK39 is associated with endothelial dysfunction and inflammatory activation under hypoxic conditions in moyamoya disease

**DOI:** 10.3389/fnhum.2026.1854099

**Published:** 2026-10-14

**Authors:** Rui Liang, Zhenyu Zhou, Yaoren Chang, Xun Ye, Youjia Tang, Shihao He, Yuanli Zhao

**Affiliations:** 1 Jiujiang City Key Laboratory of Cell Therapy, The First Hospital of Jiujiang City, Jiujiang, China; 2 Department of Neurosurgery, The First Hospital of Jiujiang City, Jiujiang, China; 3 Department of Neurosurgery, Beijing Tiantan Hospital, Capital Medical University, Beijing, China; 4 China National Clinical Research Center for Neurological Diseases, Beijing, China; 5 Department of Neurosurgery, Peking Union Medical College Hospital, Chinese Academy of Medical Sciences and Peking Union Medical College, Beijing, China; 6 Department of Pathology, Johns Hopkins School of Medicine, Baltimore, MD, United States

**Keywords:** acetylation-related genes, endothelial dysfunction, immune, moyamoya disease, pathogenesis

## Abstract

**Objective:**

Moyamoya disease (MMD) is a cerebrovascular disease whose mechanism relatively unknown and can cause vascular occlusion and hyperplasia. This study aimed to explore molecular heterogeneity associated with the expression of acetylation-related genes in MMD and to identify candidate genes potentially involved in vascular endothelial dysfunction.

**Methods:**

Public MMD transcriptomic datasets were analyzed using a predefined acetylation-regulatory gene set to identify expression clusters and prioritize cluster-associated candidate genes. Functional enrichment, immune-infiltration, and predicted miRNA–mRNA network analyses were performed to characterize candidate-associated molecular features. Serum STK39 and MTMR12 concentrations were assessed by ELISA in a preliminary study of three patients with MMD and three healthy controls. STK39 was further investigated in human brain microvascular endothelial cells (HBMECs) using siRNA-mediated knockdown under normoxic and hypoxic conditions, with Trichostatin A (TSA) treatment included as an exploratory pharmacological intervention.

**Results:**

Consensus clustering was used to classify MMD into two subtypes, and five key subtype-related genes were identified: STK39, NEO1, PKNOX1, GOLT1B, and MTMR12. In the validation cohort, serum STK39 and MTMR12 levels were significantly decreased in patients with MMD. *In vitro*, STK39 knockdown promoted HBMEC migration, inhibited proliferation, and increased the expression of HIF-1α, p-STAT3, cleaved Caspase-3, IL-6, CCL2, and VCAM-1 under hypoxic conditions, whereas TSA treatment partially reversed these changes.

**Conclusion:**

Acetylation-related genes are closely associated with the molecular heterogeneity of MMD. Among them, STK39 appears to participate in hypoxia-related endothelial dysfunction and inflammatory activation. These findings provide new insight into the role of acetylation-related pathways in MMD pathogenesis and suggest potential targets for future mechanistic studies.

## Graphical Abstract

**Figure:**
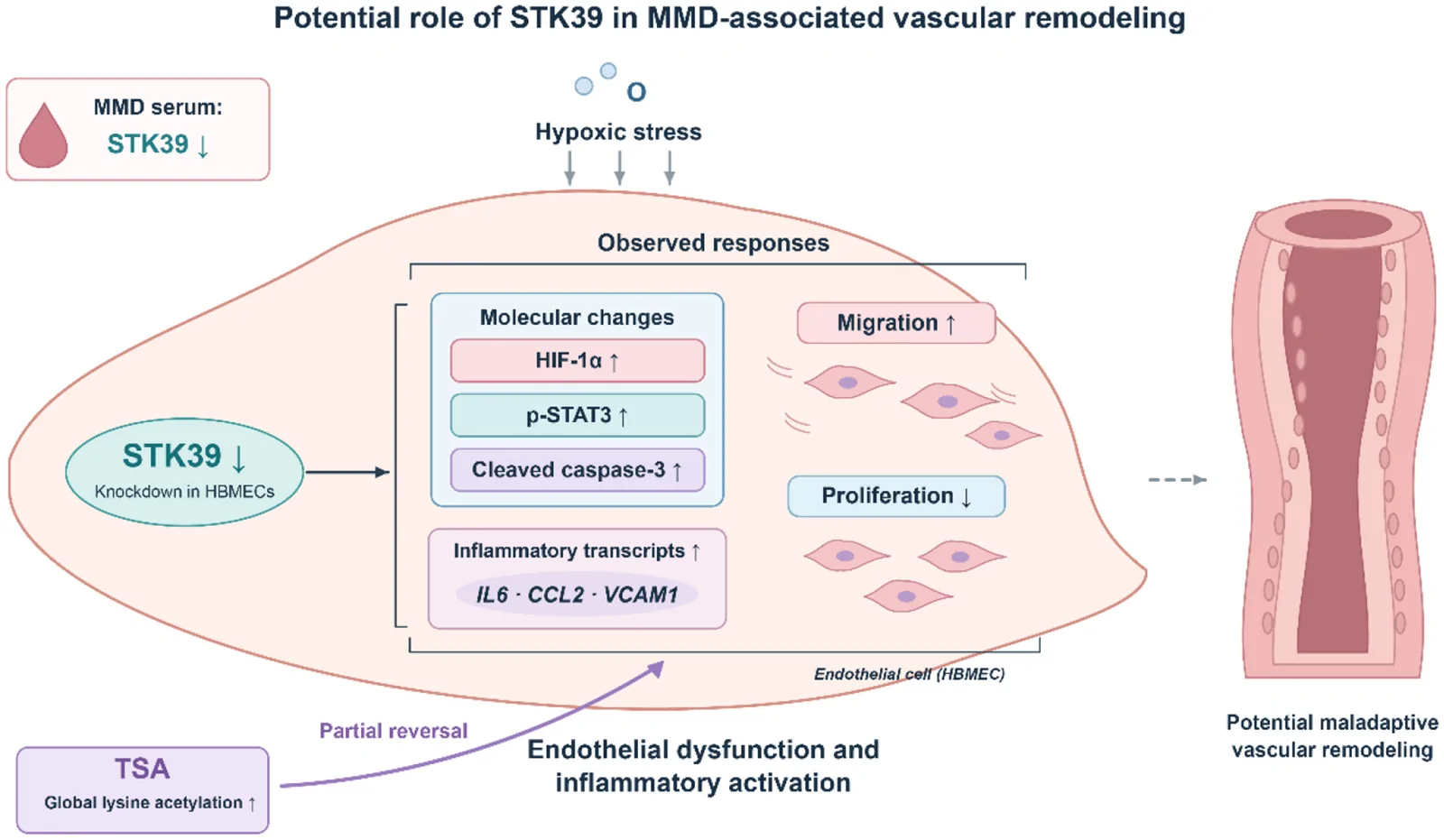

## Introduction

Moyamoya disease (MMD) is a rare chronic cerebrovascular disease. In the MMD, the cerebral ischemia caused by terminal occlusion of the internal carotid artery eventually leads to abnormal proliferation of intracranial blood vessels (He et al., 2025a). The main clinical symptoms of moyamoya disease are recurrent stroke and cognitive impairment, which cause various complications, such as aphasia, paralysis, and headache (Gonzalez et al., 2023; He et al., 2020) However, effective MMD diagnosis and treatment are limited as the pathogenesis of the disease is unknown. Recently, several studies have indicated links between vascular smooth muscle cells (VSMC) in MMD pathogenesis (Ihara et al., 2022; Guo et al., 2009).

Although RNF213 is the best-established susceptibility gene for MMD, emerging evidence indicates that the molecular pathology of MMD extends beyond a single genetic determinant. Experimental loss of RNF213 has been shown to promote pathological angiogenesis by activating YAP/TAZ–VEGFR2 signaling, linking genetic susceptibility to endothelial dysfunction and abnormal vascular remodeling (Ye et al., 2023). Complementary multi-omics studies have further revealed substantial molecular heterogeneity in MMD. Circulating small-extracellular-vesicle RNA profiling identified dysregulated RNAs associated with immune responses, endothelial dysfunction, and angiogenesis (He et al., 2023). Proteomic analysis of ischemic and hemorrhagic MMD identified differential WNT4 expression and showed that WNT4 overexpression reduced α-tubulin acetylation and microtubule stability in vascular smooth-muscle cells (He et al., 2024). More recently, DNA-methylation profiling implicated STAB1-associated epigenetic alterations in endothelial dysfunction and endothelial–immune-cell interactions in MMD (He et al., 2025b). Collectively, these studies suggest that MMD involves interacting genetic, transcriptomic, metabolic, proteomic, and epigenetic abnormalities. However, acetylation-associated gene-expression patterns in MMD remain insufficiently characterized, providing the rationale for the present exploratory investigation.

Post-translational modification (PTM), as a means of chemical modifications, regulate the function, and activity of proteins (Bradley, 2022; Yu et al., 2025). Several studies have reported the irreplaceable role of post-translational modifications in vascular diseases, including acetylation modifications in cellular autophagy, which affect vascular aging (Sun et al., 2021); ubiquitination and deubiquitination are associated with atherosclerosis, abdominal aortic aneurysm, angiogenesis, and hypertension (Wang et al., 2019; Niu et al., 2025); aberrant glycosylation of vascular calcification regulators contributes to atherosclerosis (Masbuchin et al., 2021). The acetylation, a type of PTM, is an important factor in vascular diseases development. Increased FOXO3a acetylation may promote thoracic aortic aneurysms development (Du et al., 2022). Among the mechanistic studies of moyamoya disease, the mechanisms of vascular cell abnormalities are of considerable interest. A previous study on childhood MMD found that Panobinostat enhances the vascularization ability of endothelial colony-forming cells by inhibiting histone deacetylase. During the above pathological process, Panobinostat was involved in regulating the acetylation of fretinaldehyde dehydrogenase 2. Therefore, we speculate that acetylation and acetylation-related genes may have a potential and meaningful role in MMD (Jangra et al., 2019). The focus on acetylation-related genes stems from their critical function in regulating vascular development, inflammatory responses, and cellular activities, especially in conditions that may involve abnormal vascular pathological mechanisms (Andjelkovic et al., 2022). While research on acetylation-related genes in MMD is limited, the important role of these genes in other vascular disorders suggests their potential relevance to MMD.

In this study, we used a predefined set of acetylation-regulatory genes from a previous study to explore gene-expression heterogeneity in public MMD datasets. The acetylation-related genes were extracted from previous studies (Hu et al., 2019). We then screened for candidate genes associated with the resulting expression clusters and with MMD–control differences. Candidate proteins were evaluated in serum, and STK39 was further investigated in HBMECs under normoxic and hypoxic conditions. This exploratory framework was designed to prioritize candidate genes for functional investigation and did not directly test whether STK39 is regulated by acetylation. Finally, serum STK39 and MTMR12 concentrations were assessed by Enzyme-linked immunosorbent assay (ELISA) to obtain preliminary supporting evidence regarding the biological relevance of these candidates. This offers new perspectives of the molecular mechanisms underlying MMD pathogenesis.

## Methods

### Data acquisition

The overall study design is summarized in Figure 1. Publicly available gene-expression datasets related to MMD were identified in the Gene Expression Omnibus (GEO) database using the search term “moyamoya disease,” with the organism restricted to *Homo sapiens* and datasets containing fewer than six samples excluded. Three GEO series, GSE189993, GSE157628, and GSE141024, met the initial eligibility criteria. All three series were bulk gene-expression microarray datasets generated using the Agilent-039494 SurePrint G3 Human GE v2 8 × 60K Microarray platform (GPL16699), rather than RNA-sequencing datasets. GSE189993 contained middle cerebral artery arterial-wall microsamples from 21 patients with MMD and 11 non-MMD surgical controls, including six patients with intracranial aneurysms and five patients with epilepsy. GSE157628 contained middle cerebral artery arterial-wall microsamples from 11 patients with MMD and nine non-MMD surgical controls, including six patients with intracranial aneurysms and three patients with epilepsy. GSE141024 contained superficial temporal artery arterial-wall microsamples from four patients with MMD and four patients with intracranial aneurysms. GSE189993 and GSE141024 were combined for the exploratory expression analysis. GSE157628 was excluded because it contains samples overlapping with those represented in GSE189993, thereby avoiding duplicate inclusion of these samples. Detailed information regarding the experimental platform, sample source, sample composition, original study findings, and dataset is provided in Table 1.

**Figure 1:**
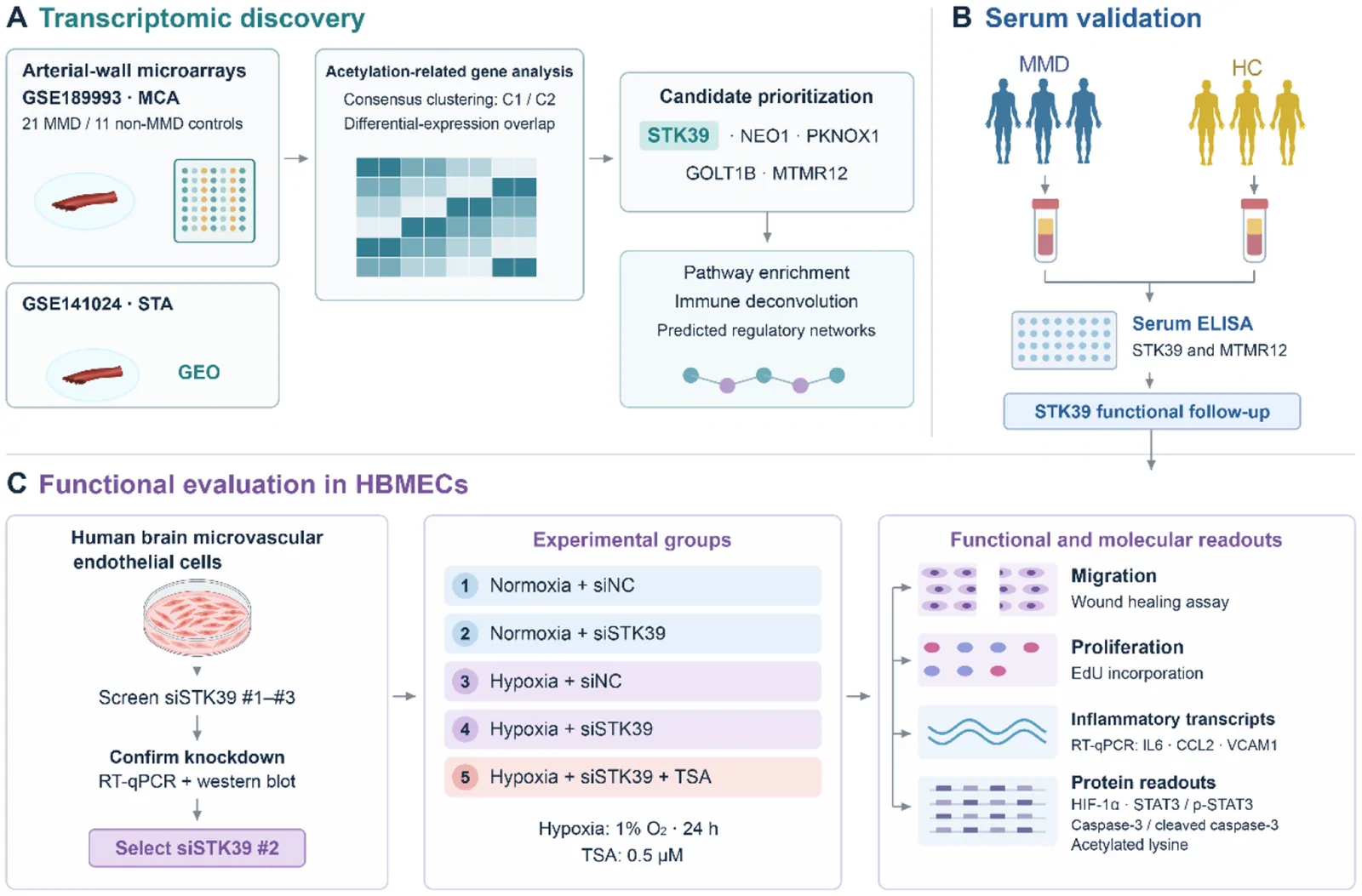
Overview of the study design and experimental workflow. **(A)** Public arterial transcriptomic datasets were analyzed to identify candidate genes associated with acetylation-related expression patterns in moyamoya disease (MMD). **(B)** Serum STK39 and MTMR12 levels were measured by ELISA in patients with MMD and healthy controls (HC). **(C)** Human brain microvascular endothelial cells (HBMECs) were subjected to STK39 knockdown under normoxic or hypoxic conditions, with or without trichostatin A (TSA). Cell migration, proliferation, inflammatory transcripts, and protein markers were assessed.

**Table 1:** Characteristics of publicly available gene-expression datasets included in the study.

GEO accession	Platform and technology	Sample type	Sample size	Major findings of the original study
GSE189993	Expression profiling by array (bulk RNA); Agilent-039494 SurePrint G3 Human GE v2 8 × 60 K Microarray, GPL16699; not RNA sequencing.	Surgically collected microsamples of middle cerebral artery (MCA) arterial wall.	32 samples: 21 MMD and 11 non-MMD controls (6 intracranial aneurysm and 5 epilepsy controls).	The original analysis identified 308 differentially expressed long noncoding RNAs (306 upregulated and 2 downregulated). Enrichment analyses implicated antibacterial humoral responses, T-cell receptor signaling, cytokine production, and branching involved in blood-vessel morphogenesis; selected findings were supported by quantitative PCR.
GSE157628	Expression profiling by array (bulk RNA); Agilent-039494 SurePrint G3 Human GE v2 8 × 60 K Microarray, GPL16699; not RNA sequencing.	Surgically collected microsamples of middle cerebral artery (MCA) arterial wall.	20 samples: 11 MMD and 9 non-MMD controls (6 intracranial aneurysm and 3 epilepsy controls).	The original study identified 62 upregulated and 26 downregulated genes in MMD MCA specimens. Immune-related pathways, including antigen processing and presentation, dendritic-cell, cytokine, and interleukin-12 pathways, were enriched, whereas oxidative phosphorylation and DNA-repair pathways were reduced. Decreased TSPAN2 and RHOQ expression was confirmed by quantitative PCR.
GSE141024	Expression profiling by array (bulk RNA); Agilent-039494 SurePrint G3 Human GE v2 8 × 60 K Microarray, GPL16699; not RNA sequencing.	Surgically collected microsamples of superficial temporal artery (STA) arterial wall; this series does not contain MCA samples.	8 samples: 4 MMD and 4 non-MMD controls (intracranial aneurysm controls).	GSE141024 is the STA component of the GSE141025 parent arterial-wall transcriptomic series. The parent series reported upregulation of inflammatory-response genes, including TLR2 and SYK, and reduced collagen-related expression, including COL15A1.

A preliminary serum study included six participants, comprising three patients with MMD and three healthy controls. Patients with MMD were diagnosed according to established diagnostic criteria (Research Committee on the Pathology and Treatment of Spontaneous Occlusion of the Circle of Willis; Health Labour Sciences Research Grant for Research on Measures for Intractable Diseases, 2012). Peripheral blood samples were collected for exploratory ELISA assessment of serum STK39 and MTMR12 concentrations. Informed written consent was obtained from all participants. All patient information are provided in Supplementary Tables S1, S3. The acetylation-related genes are listed in Supplementary Table S2. Written informed consent was obtained from all participants. This study was approved by the Institutional Ethics Committee of Peking Union Medical College Hospital, Beijing, China (I-24YSB0160).

### Consensus clustering based on acetylation-regulatory gene expression

Acetylated subtypes of moyamoya disease were clustered by a consistency clustering method with 50 iterations used for clustering. The iteration process encompasses 90% of the samples in each cycle. The optimal number of clusters was determined based on the cumulative distribution function curve of the consistency score and the characteristics observed in the heat map representation of the consistency matrix. Following identification of the C1 and C2 acetylation-related expression clusters, transcriptome-wide differential expression analysis was performed between the two groups. The purpose of this comparison was to determine whether the clusters defined by the 19 acetylation-related genes were associated with broader molecular differences beyond the genes used for clustering. Gene set enrichment analysis (GSEA), Gene set variation analysis (GSVA), and immune-infiltration analyses were subsequently performed to characterize differences in biological programs related to hypoxic injury, vascular remodeling, angiogenesis, and immune-inflammatory activity between C1 and C2.

### Functional enrichment analysis of the C1 and C2 expression clusters

The ssGSEA algorithm from the GSVA package was employed to quantify the enrichment levels of GO/KEGG pathways and assess differences in enriched pathways between subtypes. Functional categories were evaluated using GO and KEGG databases, with c2.cp.kegg.v7.5.1 and c5.go.v7.5.1 symbols serving as the background gene set.

### Batch correction and differential expression analysis

After merging the expression data from GSE189993 and GSE141024, batch adjustment was performed using ComBat in the Surrogate variable analysis (the R package used for ComBat batch adjustment is sva) sva R package, with GEO series specified as the batch variable. This adjustment was intended to reduce systematic study-associated variation potentially arising from differences in sample preparation, hybridization, scanning, and other experimental procedures. Expression patterns before and after adjustment were visualized for comparison. Because the two datasets represent different vascular tissue sources, study-associated variation may also include biological differences that cannot be fully separated from technical effects.

Following batch-effect adjustment, transcriptome-wide differential expression analysis was performed using the limma R package. All genes in the integrated expression matrix were included in the initial analysis, without prior restriction to acetylation-related genes. Linear models with empirical Bayes moderation were used to compare gene expression between the MMD and non-MMD control groups. Genes with a *P-value* < 0.05 and |log2 fold change| > 2 were considered differentially expressed. The *P-value* output from limma was used for this exploratory analysis, and no additional multiple-testing correction was applied.

The transcriptome-wide differentially expressed genes were subsequently intersected with a predefined set of 73 acetylation-regulatory genes obtained from a previous study. Genes shared by the two sets were defined as acetylation-related differentially expressed genes and were used for subsequent consensus clustering and co-expression analyses.

### Preliminary serum assessment of STK39 and MTMR12

Serum concentrations of STK39 and MTMR12 were measured by ELISA in three patients with MMD and three healthy controls. These were the only two candidate proteins assessed in the serum study; NEO1, PKNOX1, and GOLT1B were not measured. Prepare all reagents according to the instructions and prepare standard products. Add 100 μL of sample, standard or blank dilution reagent per well. Cover with rubber strips and incubate at 37 °C for 60 min. Discard the liquid. Add 100 μL of working liquid A to each well. Cover with new strips and incubate at 37 °C for 1 h. Discard the liquid. Inject 350 μL Wash Buffer into each well. Let stand for 1 min. Shake off the washing liquid. Place on absorbent paper to dry. Repeat 3 times. Add 100 μL of working liquid B to each well. Cover with new strips and incubate at 37 °C for 30 min. Repeat the above cleaning operation. Add 90 μL 3,3′,5,5′-Tetramethylbenzidine (ELISA colorimetric substrate) (TMB) substrate solution per well. Incubate at 37 °C for 20 min without light. Add 50 μL termination solution per well. Mix the liquid thoroughly. The slats were placed in the enzyme label within 30 min. OD values were measured at 450 nm.

### Cell culture

Human brain microvascular endothelial cells (HBMECs) were purchased from Shanghai Zhong Qiao Xin Zhou Biotechnology (Shanghai, China). Cells were maintained in endothelial cell medium (ECM; Shanghai Zhong Qiao Xin Zhou Biotechnology) supplemented with 5% fetal bovine serum (FBS), 1% endothelial cell growth supplement (ECGS), 100 U/mL penicillin, and 100 μg/mL streptomycin. Cells were cultured in a humidified incubator at 37 °C with 5% CO_2_. When cell confluence reached approximately 90%, cells were washed twice with Phosphate-buffered saline (PBS) and dissociated using 0.25% trypsin-0.02% Ethylenediaminetetraacetic acid (EDTA). Digestion was terminated with complete medium, and cells were collected by centrifugation at 800 rpm for 5 min at 4°C and then resuspended for subsequent experiments.

### siRNA transfection

HBMECs were seeded in 6-well plates at approximately 2 × 105 cells/well 1 day before transfection and cultured in antibiotic-free medium for 24 h until reaching 70%−90% confluence. Before transfection, cells were serum-starved for 1–2 h and transfected in serum-free Opti-MEM medium using Lipofectamine 3000 (Thermo Fisher Scientific, Pittsburgh, PA, USA) according to the experimental protocol.

Three siRNAs targeting STK39 and a negative control siRNA were synthesized by Nanjing Bion Biotechnology Co., Ltd., Jiangsu, China.

Their sense and antisense sequences are provided in Table 2.

**Table 2:** Sequences of siRNAs used for STK39 knockdown.

siRNA	Sense sequence (5^′^-3^′^)	Antisense sequence (5^′^-3^′^)
siNC	UUCUCCGAACGUGUCACGUTT	ACGUGACACGUUCGGAGAATT
siSTK39#1	GGGUGAGGAUGGUUCAGUACA	UACUGAACCAUCCUCACCCAG
siSTK39#2	GCAAGAACGUGUAGCAAUAAA	UAUUGCUACACGUUCUUGCCU
siSTK39#3	GAGAGUACCUGAUUGAGAAGC	UUCUCAAUCAGGUACUCUCUG

For transfection, Lipofectamine 3000 and siRNA were separately diluted in serum-free medium, incubated for 5 min at room temperature, then gently mixed and incubated for 20 min to allow complex formation. The transfection mixture was added to cells and incubated for 6 h, after which the medium was replaced with fresh complete medium. Transfection efficiency was verified by western blotting, and siSTK39#2 was selected for subsequent experiments.

### Experimental design and treatment groups

HBMECs were assigned to the following five groups (Table 3):

(1) Normoxia + siNC, cells transfected with negative control siRNA and cultured under normoxic conditions for 24 h;
(2) Normoxia + siSTK39, cells transfected with STK39 siRNA and cultured under normoxic conditions for 24 h;
(3) Hypoxia + siNC, cells transfected with negative control siRNA and cultured under hypoxic conditions for 24 h;
(4) Hypoxia + siSTK39, cells transfected with STK39 siRNA and cultured under hypoxic conditions for 24 h; and
(5) Hypoxia + siSTK39 + TSA, cells transfected with STK39 siRNA and cultured under hypoxic conditions for 24 h in medium containing trichostatin A (TSA) at a final concentration of 0.5 μM.

**Table 3:** Experimental design and treatment groups in HBMECs.

Group	Condition	siRNA	TSA (μM)	Exposure
Normoxia + siNC	Normoxia	siNC	0	24 h
Normoxia + siSTK39	Normoxia	siSTK39#2	0	24 h
Hypoxia + siNC	Hypoxia	siNC	0	24 h
Hypoxia + siSTK39	Hypoxia	siSTK39#2	0	24 h
Hypoxia + siSTK39 + TSA	Hypoxia	siSTK39#2	0.5	24 h

HBMECs, human brain microvascular endothelial cells; TSA, trichostatin A; Normoxia: 5% CO_2_ and 95% air. Hypoxia: 1% O_2_, 5% CO_2_, and 94% N_2_. Culture temperature: 37°C. Exposure denotes the post-transfection treatment period, not transfection duration. A TSA concentration of 0 means no TSA and does not establish vehicle matching.

Global lysine acetylation was examined only in the Hypoxia + siSTK39 and Hypoxia + siSTK39 + TSA groups. Three independent biological replicates were reported.

Normoxic culture conditions were 5% CO_2_ and 95% air, whereas hypoxic culture conditions were 1% O_2_, 5% CO_2_, and 94% N_2_. After treatment, cell proliferation was evaluated by EdU assay, migration by wound healing assay, mRNA expression of IL-6, CCL2, and VCAM-1 by RT-qPCR, and protein expression of HIF-1α, STAT3, p-STAT3, Caspase-3, cleaved Caspase-3, and Acetylated-Lysine by western blotting. Acetylated-Lysine was specifically examined in the Hypoxia + siSTK39 and Hypoxia + siSTK39 + TSA groups.

### Hypoxic exposure, STK39 knockdown, and TSA treatment

To model the hypoxic stress associated with chronic cerebral hypoperfusion in MMD, human brain microvascular endothelial cells (HBMECs) were transfected with either a non-specific negative-control siRNA (siNC) or an siRNA targeting STK39 (siSTK39) and subsequently assigned to five experimental groups. The Normoxia + siNC and Normoxia + siSTK39 groups were cultured under standard normoxic conditions (5% CO_2_ and 95% air) for 24 h. The Hypoxia + siNC and Hypoxia + siSTK39 groups were cultured under hypoxic conditions (1% O_2_, 5% CO_2_, and 94% N_2_) for 24 h. For the Hypoxia + siSTK39 + TSA group, siSTK39-transfected HBMECs were exposed to the same hypoxic conditions for 24 h in medium containing the histone deacetylase inhibitor trichostatin A (TSA; final concentration, 0.5 μM). TSA was included as an exploratory pharmacological intervention to assess its effects on cellular phenotypes following STK39 knockdown under hypoxic conditions. Global lysine acetylation was measured as a separate readout of TSA treatment; this design did not directly test acetylation-dependent regulation of STK39.

Following the 24-h exposure, cell proliferation was assessed using the 5-ethynyl-2′-deoxyuridine (EdU) incorporation assay, and cell migration was evaluated using a scratch-wound assay. The mRNA expression levels of IL6, CCL2, and VCAM1 were measured by quantitative real-time polymerase chain reaction. Protein levels of HIF-1α, STAT3, phosphorylated STAT3, caspase-3, and cleaved caspase-3 were evaluated by western blotting. Global lysine acetylation was additionally assessed by western blotting in the Hypoxia + siSTK39 and Hypoxia + siSTK39 + TSA groups. All experiments were performed using three independent biological replicates.

### Western blot analysis

For total protein extraction, HBMECs were seeded in 6-well plates at 5 × 105 cells/well and subjected to the indicated treatments after reaching approximately 90% confluence. Cells were collected, washed twice with ice-cold PBS, and lysed in Radioimmunoprecipitation assay (RIPA) buffer containing Phenylmethylsulfonyl fluoride (PMSF). Lysates were centrifuged at 12,000 rpm for 5 min at 4 °C, and the supernatants were collected for protein analysis. Protein concentrations were determined using a Bicinchoninic acid (BCA) protein assay kit. Samples were mixed with 5 × loading buffer and boiled for 10 min before electrophoresis.

Equal amounts of protein (60 μg per lane) were separated by SDS-PAGE using 10% resolving gels and 5% stacking gels and then transferred onto 0.2 μm Polyvinylidene difluoride (PVDF) membranes. Membranes were activated in methanol, equilibrated in transfer buffer, and blocked with 5% non-fat milk in Tris-buffered saline with Tween 20 (TBST) for 1 h at room temperature. Membranes were incubated overnight at 4 °C with the following primary antibodies: STK39 (1:2000), HIF-1α (1:1000), STAT3 (1:2000), p-STAT3 (1:1000), Caspase-3 (1:1000), Acetylated-Lysine (1:1000), and β-actin (1:1000). After washing with TBST, membranes were incubated with Horseradish peroxidase (HRP)-conjugated goat anti-rabbit IgG or rabbit anti-mouse IgG secondary antibodies (both 1:10,000) for 1 h at room temperature. Protein bands were visualized using enhanced chemiluminescence reagents and detected using a Tanon 5200 imaging system. Densitometric analysis was performed using Image-Pro Plus 6.0, and protein expression levels were normalized to β-actin.

### RNA extraction and quantitative real-time PCR

Total RNA was extracted from treated HBMECs using TRIzol reagent (Takara, Japan) according to the manufacturer's instructions. Briefly, cells were lysed in 0.5 mL TRIzol per well, mixed with chloroform, and centrifuged at 12,000 rpm for 15 min at 4 °C. The aqueous phase was collected and RNA was precipitated with isopropanol at −20°C for 30 min. After centrifugation, the RNA pellet was washed with 75% ethanol, air-dried, and dissolved in DEPC-treated water. RNA concentration and purity were measured using a NanoDrop 2000 spectrophotometer.

For reverse transcription, 2 μL of total RNA was used to synthesize cDNA in a 20 μL reaction volume with the iScript cDNA synthesis kit (Bio-Rad, Hercules, CA, USA). The reverse transcription program was set as follows: 25 °C for 5 min, 46 °C for 20 min, and 95 °C for 1 min.

Quantitative real-time PCR was performed using TB Green Premix Ex Taq (Takara, Japan) on an ABI 7500 real-time PCR system. The 20 μL PCR reaction mixture contained 10 μL 2 × TB Green Premix, 0.4 μL forward primer, 0.4 μL reverse primer, 0.4 μL ROX reference dye, 2 μL cDNA template, and 6.8 μL nuclease-free water. Relative mRNA expression levels were calculated using the 2^−ΔΔCt^ method with β-actin as the internal control.

The primer sequences used for RT-qPCR are listed in Table 4.

**Table 4:** Primer sequences used for RT-qPCR.

Gene	Forward primer (5^′^-3^′^)	Reverse primer (5^′^-3^′^)
STK39	AGCTGCAGGAGGTTATCG	CCGTTTTATTGCTACACGTT
IL6	CCACCGGGAACGAAAGAGAA	GAGAAGGCAACTGGACCGAA
CCL2	ACCCAGAAACATCCAATTCT	TCTGCACTGAGATCTTCCTA
VCAM1	AGATAGTCCACTGAATGGGA	TGGGTATACATCAGCAACTG
ACTB	CACCATTGGCAATGAGCGGTTC	AGGTCTTTGCGGATGTCCACGT

ACTB encodes β-actin and was used as the internal reference. Relative expression was calculated using the 2^−ΔΔCt^ method. IL6 and VCAM1 correspond to IL-6 and VCAM-1 in the original primer list. ^**^P < 0.01. *n* = 3.

### EdU proliferation assay

Cell proliferation was assessed using the BeyoClick™ EdU-594 Cell Proliferation Kit (Beyotime, Shanghai, China). HBMECs were seeded into 96-well plates at a density of 1 × 104 cells/well and allowed to attach overnight before treatment. A 2 × EdU working solution was prepared by diluting 10 mM EdU at 1:500 in culture medium to a final concentration of 20 μM. Equal volumes of the 2 × EdU solution were added to each well to achieve a final EdU concentration of 10 μM, followed by incubation for 2 h.

After labeling, cells were fixed with 4% paraformaldehyde for 15 min at room temperature, washed with PBS, permeabilized with 0.3% Triton X-100 for 15 min, and incubated with Click reaction solution for 30 min in the dark at room temperature. For nuclear staining, Hoechst 33342 was diluted 1:1,000 in PBS to prepare the working solution. Each well was incubated with 100 μL of the 1 × Hoechst 33342 working solution for 10 min at room temperature in the dark. The working solution was then removed, and the cells were washed three times with PBS for 5 min each. Images were acquired under a fluorescence microscope at × 200 magnification.

### Wound healing assay

Cell migration was evaluated using a wound healing assay. HBMECs in the logarithmic growth phase were seeded into 6-well plates at a density of 6 × 105 cells/mL, with 2 mL cell suspension per well, and cultured for 24 h until a confluent monolayer formed. Straight reference lines were drawn on the back of the wells before seeding. A sterile 200 μL pipette tip was used to create a scratch perpendicular to the reference lines. Detached cells were removed by washing three times with PBS, and cells were then cultured in serum-free medium. Images were captured at 0 h and 24 h under an inverted microscope at × 100 magnification, ensuring that the scratch area remained centered and vertically aligned. The wound area was measured at 0 h and 24 h after scratching. Wound closure for each sample was calculated as [(wound area at 0 h – wound area at 24 h)/wound area at 0 h] × 100%. Relative wound closure was calculated by normalizing each sample's wound-closure percentage to the mean value of the Normoxia + siNC group, which was set to 100%.

### Reagents and antibodies

Trichostatin A (TSA) was purchased from MedChemExpress (Shanghai, China). FBS was obtained from HyClone (Logan, UT, USA). Lipofectamine 3000 was purchased from Thermo Fisher Scientific (Pittsburgh, PA, USA). Anti-STK39 and anti-STAT3 antibodies were obtained from Proteintech (Hubei, China). Anti-HIF-1α, anti-p-STAT3, anti-Caspase-3, and anti-Acetylated-Lysine antibodies were obtained from Cell Signaling Technology (MA, USA). Anti-β-actin and HRP-conjugated secondary antibodies were purchased from Abcam (Cambridge, UK). Other reagents were obtained from the sources described in the experimental report.

### Analysis of immune cell infiltration

The CIBERSORT algorithm, consisting of 547 biomarkers and 22 human immune cell phenotypes, was employed in this study to assess the immune cell phenotypes within microenvironments. Patient data were subjected to analysis using CIBERSORT for evaluating the relative proportions of immune cells and examining the correlation between genes and immune cells.

### miRNA network architecture

The miRNA (MicroRNAs) is a type of non-coding RNA that can regulate mRNA. The analysis focused on the examination of miRNAs derived from key genes obtained through the miRcode database, with regards to their impact on the transcription or degradation of other genes. Relevant miRNAs were acquired from the miRcode database and cytoscape software was utilized to visualize the gene-associated miRNA networks.

### GSEA pathway enrichment analysis

The difference of signaling pathway was assessed by GSEA in the acetylation-related genes low and high expression cohorts. The gene set annotated for the subtype pathway was obtained from the MsigDB database. Subtype-specific differential expression analysis of the pathway was conducted, and gene sets showing significant enrichment (*P* < 0.05) were ranked based on their consistency score. The GSEA software (Java version) was utilized to compare KEGG signaling pathways between the low and high expression groups.

### GSVA enrichment analysis

The Gene set variation analysis (GSVA) is a method that does not rely on specific assumptions or supervision to assess the enrichment of gene sets in transcriptome data. GSVA employs comprehensive scores to convert genetic variations into pathway differences, enabling the evaluation of biological functions based on these pathway distinctions. In this study, we obtained gene datasets from the Molecular signatures database and utilized the GSVA algorithm to analyze their pathways, aiming to identify potential changes in biological functionality across different datasets.

### Analysis of transcriptional regulation of key genes

In this study, the prediction of potential transcription factors was conducted using the R-package “RcisTarget.” The functionality of RcisTarget relies on motif analysis. The normalized enrichment score (NES) for Motifs is determined based on the overall number of motifs present in the database. The annotation file is derived from gene sequence and its similarity. To calculate the area under the curve (AUC) for each pair of motif-motifs, a recovery curve is generated using gene set-based sequence analysis. Subsequently, NES values were computed for all motifs based on AUC scores obtained from gene datasets.

### Statistical analysis

Statistical analysis and plotting of the cellular experimental data were performed using GraphPad Prism 9 (version 9.4.0). Figure panels were assembled using Adobe Illustrator 2026. Data are presented as the mean ± SD from three independently performed experiments. Each independent experiment constituted one experimental replicate. Between-group comparisons were performed using one-way ANOVA followed by Tukey's multiple-comparisons test. For pairwise comparisons, a Tukey-adjusted *P-value* < 0.05 was considered statistically significant.

## Result

### Inclusion and differential analysis of gene data related to MMD and acetylation

The exploratory expression analysis combined 32 middle cerebral artery samples from GSE189993, comprising 21 MMD and 11 non-MMD surgical control samples, with eight superficial temporal artery samples from GSE141024, comprising four MMD and four non-MMD control samples. GSE157628 was excluded to avoid duplicate inclusion of overlapping samples. The merged expression matrix was adjusted using ComBat in the sva package. The results demonstrated that the interchip batch effect was reduced after SVA algorithm correction (Supplementary Figures S1A, B). Acetylation-related genes were obtained from a previous study (Hu et al., 2019). Acetylation-related genes expression in controls and MMD patients was analyzed. The results demonstrated that 19 acetylation-related genes, including BAZ1B, BRD3, and BRD7, were differentially expressed in the control and MMD patient groups. After the batch effect was eliminated, the acetylation correlation in the dataset was analyzed and gene expression levels were mapped with standardized values (Supplementary Figure S1C). The 34 transcriptome-wide differentially expressed genes were subsequently intersected with the predefined set of 73 acetylation-regulatory genes. This analysis identified 19 acetylation-related differentially expressed genes, including BAZ1B, BRD3, and BRD7. These 19 genes were used for subsequent consensus clustering and co-expression analyses (Supplementary Figures S1C, D). Correlation analysis of 19 acetylation-related genes in the control and MMD patient cohorts was performed (Figures 2A, B).

**Figure 2:**
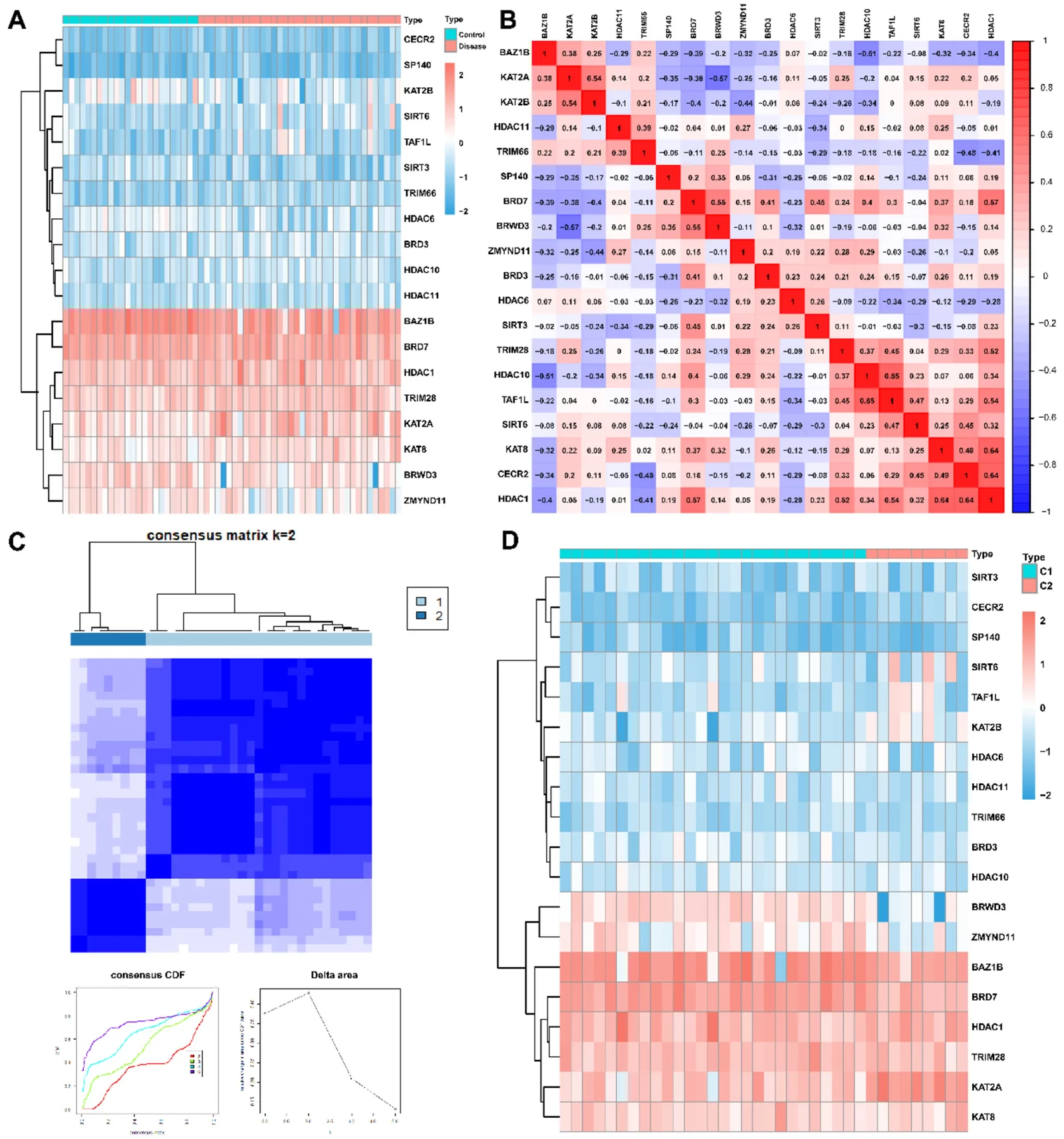
Association analysis of acetylation-related genes and molecular typing. **(A)** Analysis of differences in acetylation-related genes between the control and disease groups (blue: control; red: disease group; red represents upregulated gene expression levels; darker colors represent more upregulated genes). Blue represents the downregulation of gene expression levels, and darker colors represent more downregulation. **(B)** Correlation analysis of acetylation-related genes in the disease groups (Red indicates a positive correlation, blue indicates a negative correlation, and darker colors indicate a deeper correlation). **(C)** When *k* = 2, disease groups are molecularly classified according to the level of acetylation-related genes (Abscissa: light blue denotes C1; C2: dark blue). **(D)** Expression levels of acetylation-related genes in the two mmd acetylation isoforms C1 and C2 (Abscissa: blue represents C1; Red represents C2; Ordinate: intersection of 19 acetylation-related genes and MMD genes; Red represents upregulated gene expression levels, and darker colors represent more upregulated genes. Blue represents the downregulation of gene expression levels, and darker colors represent more downregulation).

### Identification of acetylation-related expression clusters in MMD

The consistency clustering method was used to classify disease groups based on the expression levels of acetylation-related genes. When *K* = 2, the difference between the two cluster was most obvious (Figure 2C). To determine whether the acetylation-related expression clusters were associated with broader biological differences, differential expression analysis was performed between C1 and C2 after cluster assignment. Therefore, MMD was divided into two cluster related to acetylation. Simultaneously, the acetylation-related genes expression levels in the two isoforms were shown as a heat map (Figure 2D).

To identify the relevant key genes, the expression profiles of C1 and C2 were analyzed using the limma package, comparing differences between the disease and control groups. The difference in genetic screening condition is: P.V < 0.05 and | logFC | > 2. In total, 472 differentially expressed genes were assessed from C1 and C2, including 92 upregulated and 380 downregulated genes (Figures 3A, B). Following batch-effect adjustment, transcriptome-wide differential expression analysis was performed between the MMD and non-MMD control groups without prior restriction to a specific gene set. Using the criteria of *P* < 0.05 and |log2 fold change| > 2, 34 transcriptome-wide differentially expressed genes were identified, including six upregulated and 28 downregulated genes (Supplementary Figure S2). Intersecting the genes differentially expressed between C1 and C2 with those differentially expressed between MMD and non-MMD controls yielded five candidate genes, STK39, NEO1, PKNOX1, GOLT1B, and MTMR12 (Figure 3C). These genes were prioritized through their association with the expression clusters, rather than through direct evidence of acetylation-dependent regulation. In exploratory comparisons by control diagnosis, STK39 and NEO1 met the within-comparison transcriptome-wide False discovery rate (FDR) threshold when the 21 MMD samples were compared with the five epilepsy controls. For STK39, the nominal *P-value* was 0.00208549 and the adjusted *P-value* was 0.0305491; the adjusted *P-value* for NEO1 was 0.0316719. None of the five candidates met the FDR threshold in the comparison with the six aneurysm controls. These additional findings were interpreted as exploratory and did not replace the primary comparison. To identify the enriched gene sets within each subtype, GSEA was conducted. The most representative pathways were selected for cluster 1 and cluster 2 for heat mapping, which revealed that different pathways were enriched for each isoform (Figures 3D, E).

**Figure 3:**
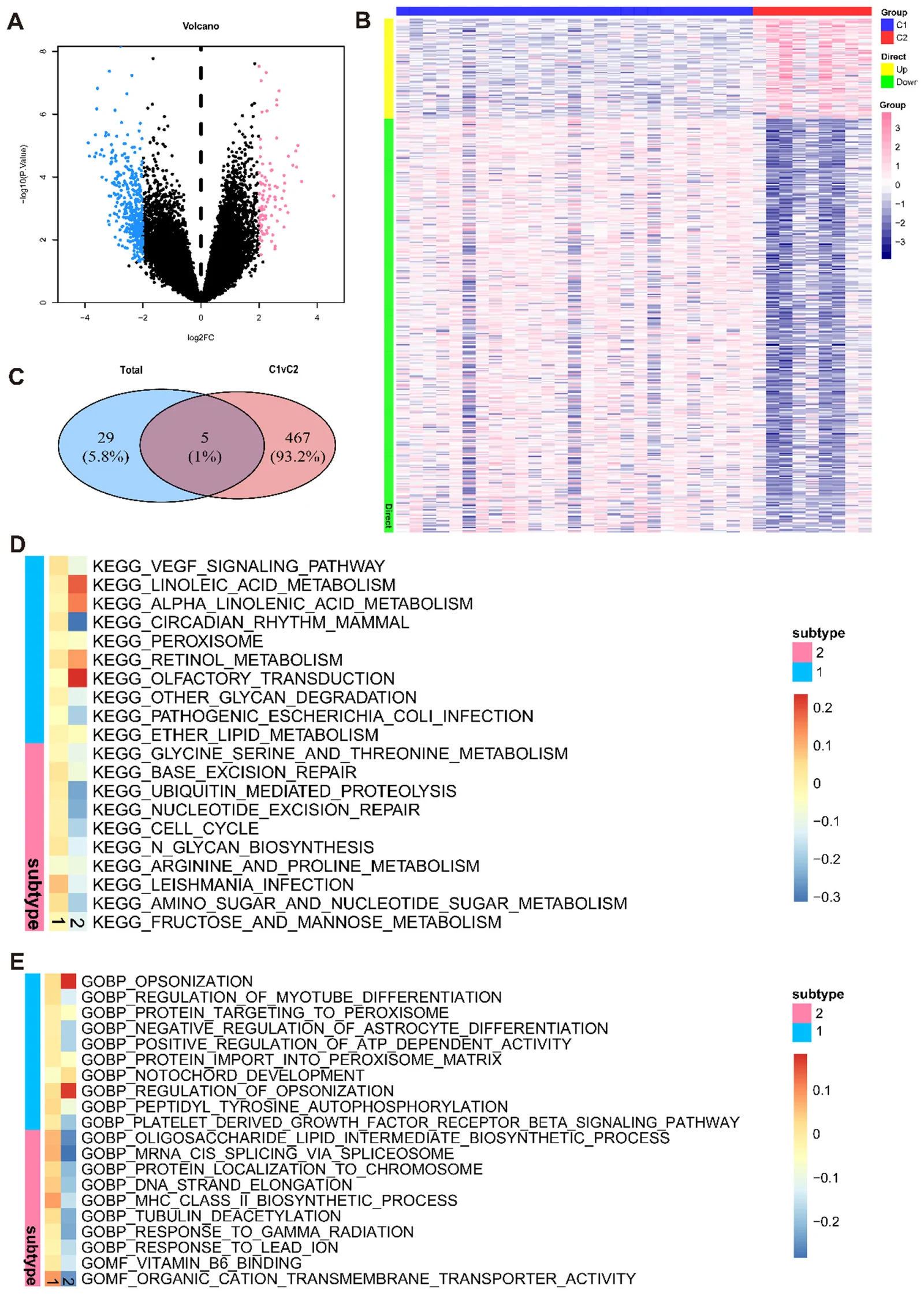
Screening of key genes and enrichment analysis of acetylated isoforms. **(A)** Intersecting genes that met the screening criteria from the differential analysis of the gene sets of the two acetylation subtypes of moyamoya disease using the limma package (Blue: downregulated genes; Red: upregulated genes; Differences in genetic screening conditions are: *P-value* < 0.05 and | logFC | > 2. A total of 472 differentially expressed genes are identified, including 92 upregulated and 380 downregulated genes). **(B)** Heat map of the differential analysis of gene sets for the two acetylation subtypes of moyamoya disease (blue: C1; Red: C2). Red represents upregulated gene expression levels and darker colors represent upregulated genes. Blue represents the downregulation of gene expression levels, and darker colors represent more downregulation. **(C)** The differentially expressed genes of the two acetylation types of moyamoya disease and the differentially expressed genes of the control and disease groups are intercrossed to obtain five key genes (Blue indicates the screened differential genes between the control and disease groups, with a total of 29 genes. Red indicates the 467 differentially expressed genes screened from groups C1 and C2. Purple from the intersection of two sets of genetic variations in genes; five are designated as key genes: STK39, NEO1, PKNOX1, GOLT1B, and MTMR12). **(D)** KEGG enrichment analysis of the two acetylation subtypes of moyamoya disease. Subtype: pink for C2 and blue for C1 (Red indicates that the pathway is upregulated, and the darker the color, the more upregulated the pathway. Blue represents the downregulation of pathways, and darker colors represent more downregulation). **(E)** GO enrichment analysis of the two acetylation subtypes of moyamoya disease. Subtype: pink for C2 and blue for C1 (Red indicates that the pathway is upregulated, and the darker the color, the more upregulated the pathway. Blue represents the downregulation of pathways, and darker colors represent more downregulation).

Expression characteristics and regulatory network of the five candidate genes. Five genes were prioritized as exploratory candidates for further biological investigation. The differences in key genes between the MMD (C1, C2) and control groups were evaluated (Figures 4A, B). The key genes were reverse predicted using by the mircode database. Eighty two miRNAs and 230 pairs of mRNA–miRNAs were obtained (Figure 4C).

**Figure 4:**
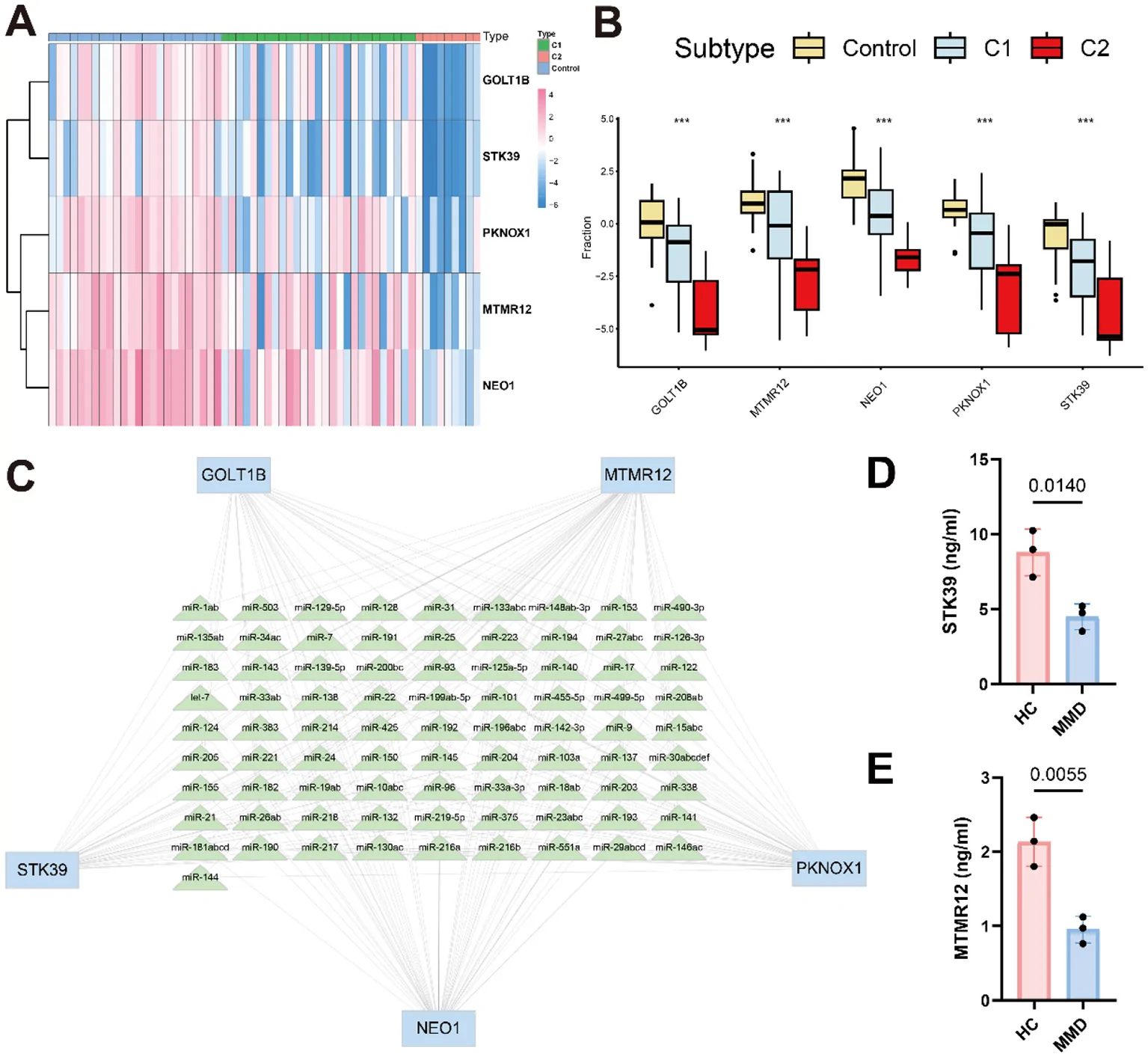
Differential analysis and regulatory network of five key genes in C1 and C2. **(A)** Analysis of differences in key genes between the control and the two acetylation subtypes of moyamoya disease (Type Green indicates C1; Red indicates C2; Blue indicates the control group. Pink represents upregulated pathways, and darker colors represent more upregulated pathways. Blue represents the downregulation of pathways, and darker colors represent more downregulation). **(B)** Expression levels of key genes in the two acetylation subtypes of moyamoya disease (Subtypes: yellow indicates the control group, C1 is blue, and C2 is red. ***P < 0.001). **(C)** Predicted miRNA–mRNA regulatory network of the five candidate genes, STK39, MTMR12, GOLT1B, PKNOX1, and NEO1. Blue rectangular nodes represent the candidate genes, green triangular nodes represent their predicted regulatory miRNAs, and gray edges indicate predicted miRNA–target interactions. The interactions were obtained using the database and screening criteria described in the Methods and visualized using Cytoscape. **(D)** The levels of STK39 in HC Serum and MMD serum were detected by ELISA kit. Results were shown as mean ± SD. *P < 0.05, **P < 0.01. Each dot represents one participant, with three participants per group. **(E)** The levels of MTMR12 in HC Serum and MMD serum were detected by ELISA kit. Results were shown as mean ± SD. *P < 0.05, **P < 0.01. Each dot represents one participant, with three participants per group.

In a preliminary serum study comprising three patients with MMD and three healthy controls, ELISA measurements showed lower serum STK39 and MTMR12 concentrations in the MMD group (*P* = 0.0140 and *P* = 0.0055, respectively; Figures 4D, E). Only these two candidate proteins were measured; NEO1, PKNOX1, and GOLT1B were not assessed. Given the small sample size, these findings were considered preliminary supporting evidence rather than independent validation of the transcriptomic candidates. The patients' clinical characteristics and sample information are presented in Supplementary Table S3.

### Validation of STK39 knockdown efficiency in HBMECs

We first screened three candidate siRNAs targeting STK39 to determine the optimal sequence for subsequent loss-of-function experiments in HBMECs (Figure 5). Western blot analysis demonstrated that STK39 protein levels were decreased after transfection with all three siRNAs, whereas siSTK39#2 showed the most prominent reduction among the tested constructs. Similarly, RT-qPCR analysis confirmed a substantial decrease in STK39 mRNA expression in the siSTK39 groups, with siSTK39#2 again showing the strongest silencing efficiency. No obvious difference was observed between the control and siNC groups. Therefore, siSTK39#2 was selected for all subsequent experiments.

**Figure 5:**
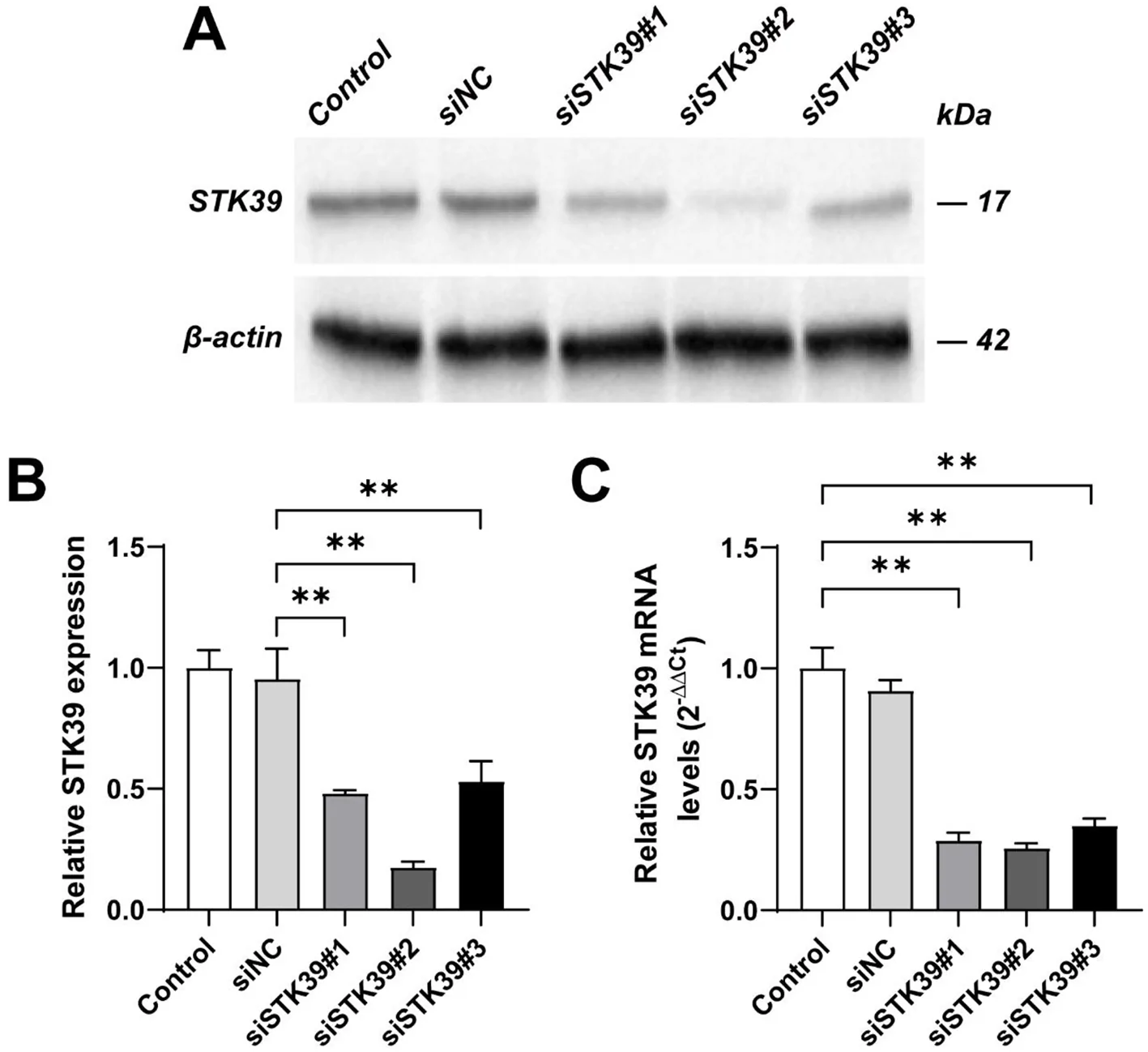
Validation of STK39 knockdown efficiency in HBMECs transfected with different siRNAs. **(A)** Representative western blot showing STK39 protein expression in HBMECs transfected with negative control siRNA (siNC) or three different STK39-targeting siRNAs (siSTK39#1, siSTK39#2, and siSTK39#3). β-actin was used as the loading control. **(B)** Quantitative analysis of STK39 protein expression normalized to β-actin. **(C)** T-qPCR analysis of STK39 mRNA expression in HBMECs after transfection with siNC or the indicated siSTK39 constructs. **P < 0.01. *n* = 3.

### STK39 knockdown promoted migration but suppressed proliferation of HBMECs under hypoxic conditions, and TSA partially reversed these effects

To evaluate the effects of STK39 knockdown on the migratory capacity of HBMECs under normoxic and hypoxic conditions, a wound healing assay was performed. Representative images are shown in Figure 5A. Under normoxic conditions, HBMECs transfected with siSTK39 displayed greater wound closure than those in the Normoxia + siNC group. Under hypoxic conditions, the migratory ability of HBMECs was further increased, and this effect was more pronounced after STK39 silencing. Quantitative analysis confirmed that the relative migration ability was significantly higher in the Normoxia + siSTK39 group than in the Normoxia + siNC group, and was further elevated in the Hypoxia + siSTK39 group compared with the Hypoxia + siNC group (Figure 6C). After TSA treatment, the enhanced migration induced by STK39 knockdown under hypoxia was significantly attenuated, although the migration level remained higher than that in the Hypoxia + siNC group (Figure 6C). This comparison was interpreted as an exploratory observation rather than evidence of STK39-specific rescue. Cell proliferation was then assessed by EdU staining. Representative fluorescence images are presented in Figure 6B. Under normoxic conditions, STK39 knockdown slightly reduced the number of EdU-positive cells compared with the Normoxia + siNC group. Hypoxic exposure markedly decreased HBMEC proliferation, and STK39 silencing caused a further reduction in proliferative activity. Consistent with the representative images, quantitative analysis showed that the cell proliferation percentage was significantly lower in the Hypoxia + siSTK39 group than in the Hypoxia + siNC group (Figure 6D). Notably, TSA treatment significantly restored the proliferation of HBMECs under hypoxic conditions after STK39 knockdown, although it did not fully recover to the level observed in the Hypoxia + siNC group (Figure 6D).

**Figure 6:**
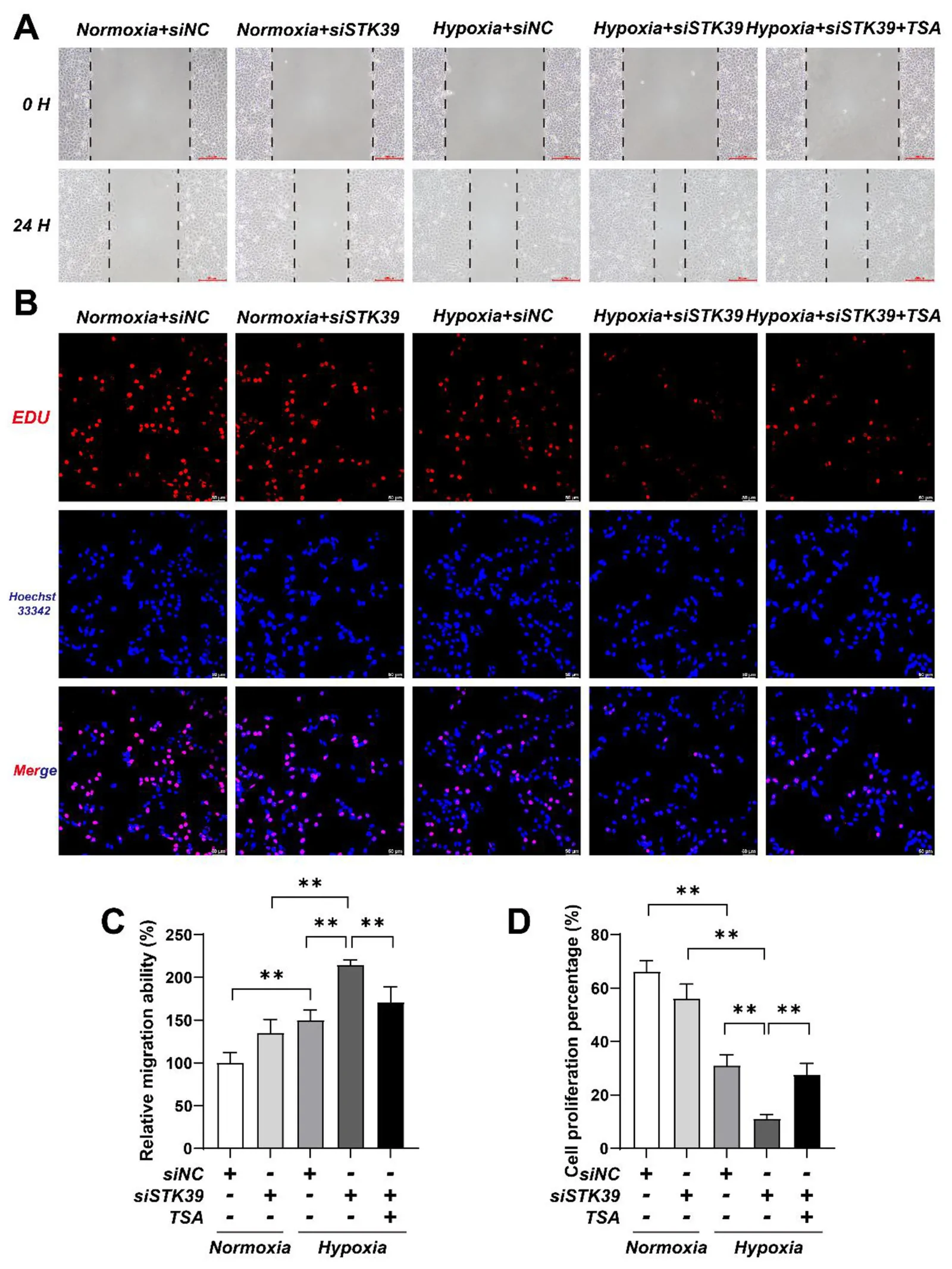
Effects of STK39 knockdown and TSA treatment on migration and proliferation of HBMECs under normoxic and hypoxic conditions. **(A)** Presentative images of the wound healing assay in HBMECs from the Normoxia + siNC, Normoxia + siSTK39, Hypoxia + siNC, Hypoxia + siSTK39, and Hypoxia + siSTK39 + TSA groups at 0 h and 24 h. **(B)** Presentative EdU staining images of HBMECs in the indicated groups. EdU-positive proliferating cells are shown in red, nuclei are counterstained with Hoechst 33342 in blue, and merged images are shown in the lower panels. **(C)** Quantitative analysis of relative migration ability based on wound healing assay results. **(D)** Quantitative analysis of cell proliferation percentage based on EdU-positive cells. STK39 knockdown enhanced migration and reduced proliferation of HBMECs under hypoxic conditions, while TSA treatment partially reversed these changes. Data are presented as mean ± SD from three independent experiments (*n* = 3). Statistical comparisons were performed using one-way ANOVA followed by Tukey's multiple-comparisons test. Asterisks indicate Tukey-adjusted *P-values* for the indicated comparisons: **P < 0.01.

### STK39 knockdown aggravated hypoxia-induced endothelial injury, and TSA partially reversed these changes

Western blot results showed that hypoxia increased the protein levels of HIF-1α, p-STAT3, and cleaved Caspase-3 in HBMECs, while total STAT3 and Caspase-3 showed no obvious change (Figures 7A, C–G). After STK39 knockdown, the expression of HIF-1α, p-STAT3, and cleaved Caspase-3 was further increased under hypoxic conditions (Figures 7A, C, E, G). TSA treatment partially reduced these changes.

**Figure 7:**
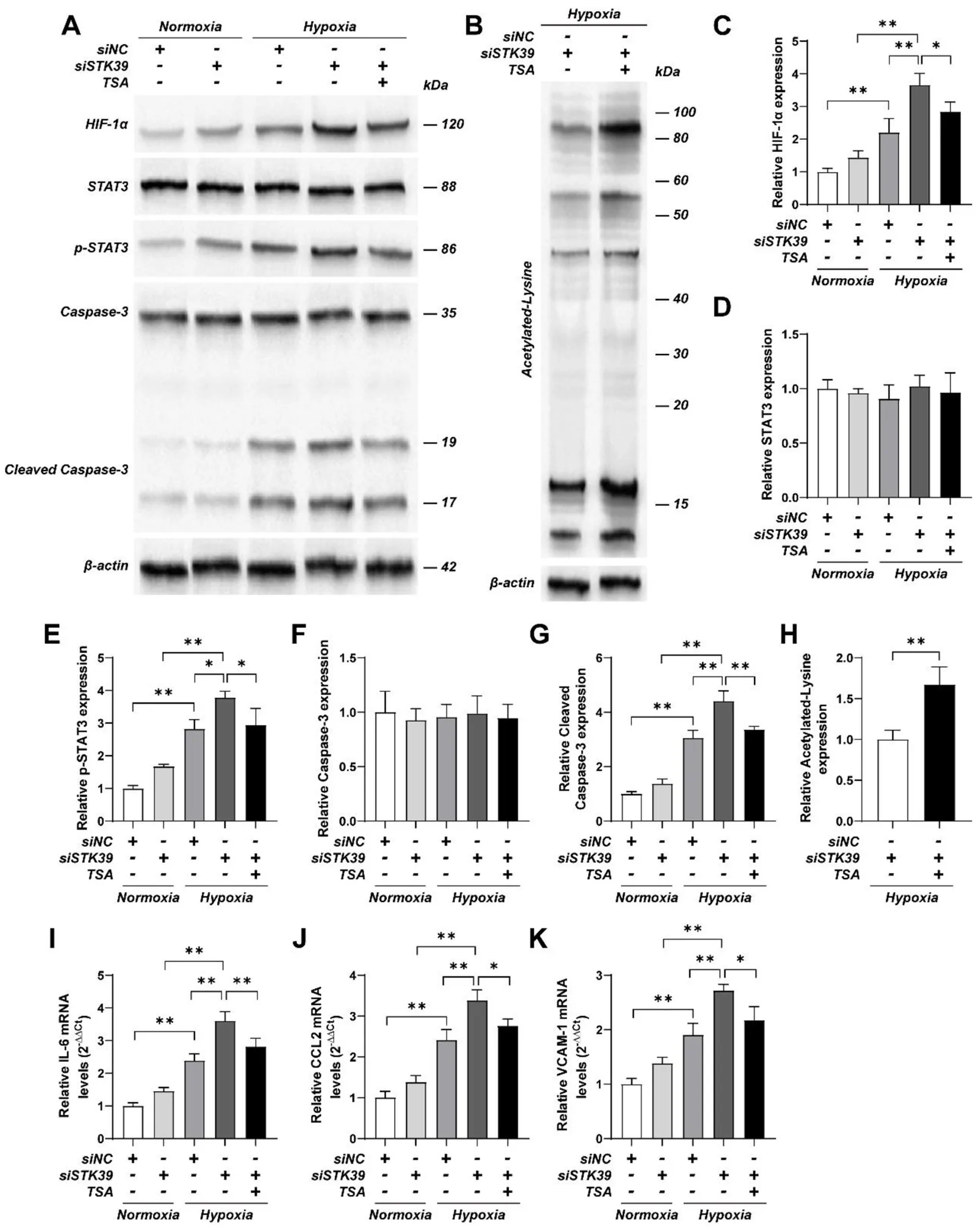
Effects of STK39 knockdown and TSA treatment on hypoxia-induced injury-related and inflammatory markers in HBMECs. **(A)** Representative western blot images showing the protein expression of HIF-1α, STAT3, p-STAT3, Caspase-3, cleaved Caspase-3, and β-actin in HBMECs from the Normoxia + siNC, Normoxia + siSTK39, Hypoxia + siNC, Hypoxia + siSTK39, and Hypoxia + siSTK39 + TSA groups. **(B)** Representative western blot images showing acetylated-lysine expression in the Hypoxia + siSTK39 and Hypoxia + siSTK39 + TSA groups. β-actin was used as the loading control. **(C–H)** Quantitative analysis of HIF-1α, STAT3, p-STAT3, Caspase-3, cleaved Caspase-3, and acetylated-lysine protein expression. **(I–K)** RT-qPCR analysis of IL-6, CCL2, and VCAM-1 mRNA expression in the indicated groups. STK39 knockdown further increased hypoxia-induced HIF-1α, p-STAT3, cleaved Caspase-3, and inflammatory factor expression, whereas TSA treatment partially reversed these changes. Data are presented as mean ± SD. *P < 0.05. **P < 0.01. *n* = 3.

Western blotting showed a higher global lysine acetylation signal in the Hypoxia + siSTK39 + TSA group than in the Hypoxia + siSTK39 group (Figures 7B, H).

RT-qPCR results further showed that hypoxia increased the mRNA levels of IL-6, CCL2, and VCAM-1, and these increases were more obvious after STK39 knockdown (Figures 7I–K). TSA treatment partly decreased the expression of these inflammatory factors (Figures 7I–K).

Together, these results suggest that STK39 knockdown aggravated hypoxia-induced endothelial injury and inflammatory activation, while TSA partially reversed these effects.

### The links between key genes and immune infiltration in MMD

Upon analyzing the relationship between the MMD subtype and immunity, the results indicated that the distribution of immune infiltration and the level of immune cells had significant changes (Figures 8A, B and Supplementary Figure S3). In addition, plasma cells, T regulatory cells (Tregs), and monocytes differ significantly between different subtypes (Figure 7B). PKNOX1 was positively correlated with neutrophil and NK cell activation and negatively correlated with resting dendritic and plasma cells. MTMR12 was positively correlated with neutrophil and NK cell activation and negatively correlated with Eosinophils and CD8 T cells (Figure 8C). The differences in immune factors between the two subtypes were obtained from the Tumor and immune system interaction database (TISIDB) database (Supplementary Figure S3). This suggests that key genes may be involved in the pathogenesis of MMD by influencing immune cells and immune factors.

**Figure 8:**
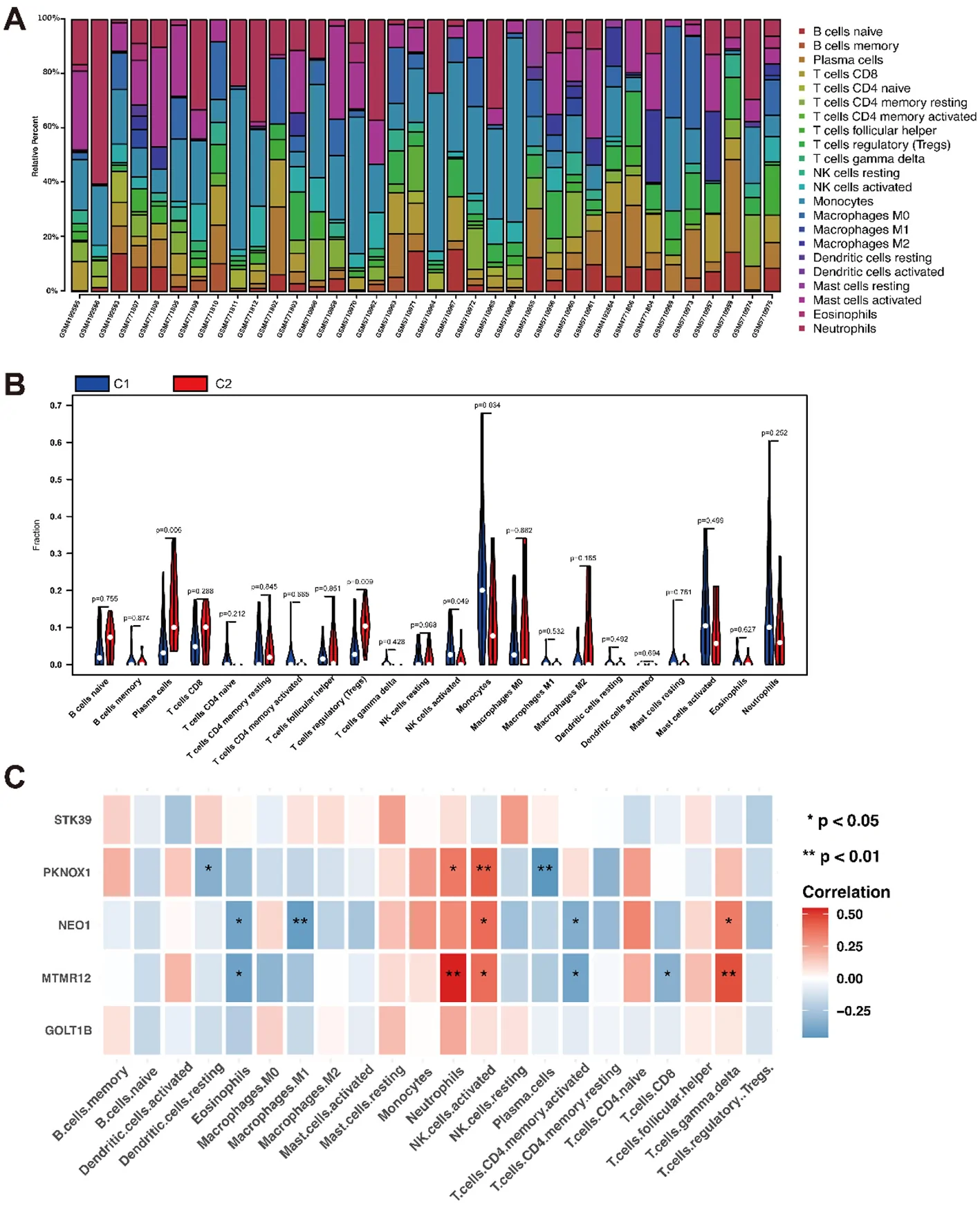
Composition of immune infiltrating cells in association with the five key genes. **(A)** Proportion of 22 immune cells in patients with moyamoya disease (Ordinate: 0%−100% represents the proportion of diverse immune cells. Abscissa: dataset number for patients with moyamoya disease). **(B)** Comparative analysis of immune cells in the two acetylation subtypes of moyamoya disease (C1 in blue and C2 in red). Ordinate: expression levels of immune cells. Abscissa: immune cell name. **(C**) Map of correlations between the five key genes and immune-infiltrating cells (Ordinate: five key genes). Abscissa: Immune cell name. *P < 0.05, **P < 0.01; the redder the color, the stronger the positive correlation; the deeper the blue color, the stronger the negative correlation.

### Discussion on specific signaling mechanisms related to the five key genes

The results of the GSEA demonstrated that STK39 enriched pathways included the lipoic acid metabolism and Proteasome and Sphingolipid signaling pathway (Figures 9A, B). The pathways enriched with NEO1 included the ErbB signaling pathway, Fanconi anemia pathway, tryptophan metabolism, and others pathways (Supplementary Figure S4A). PKNOX1 was enriched in the HIF-1 signaling pathway, mRNA surveillance pathway, and tryptophan metabolism (Supplementary Figure S4B). GOLT1B was enriched in circadian rhythm, pyruvate metabolism, tryptophan metabolism (Supplementary Figure S4C). The pathways enriched with MTMR12 included the circadian rhythm, ErbB signaling pathway, and pyruvate metabolism (Supplementary Figure S4D).

**Figure 9:**
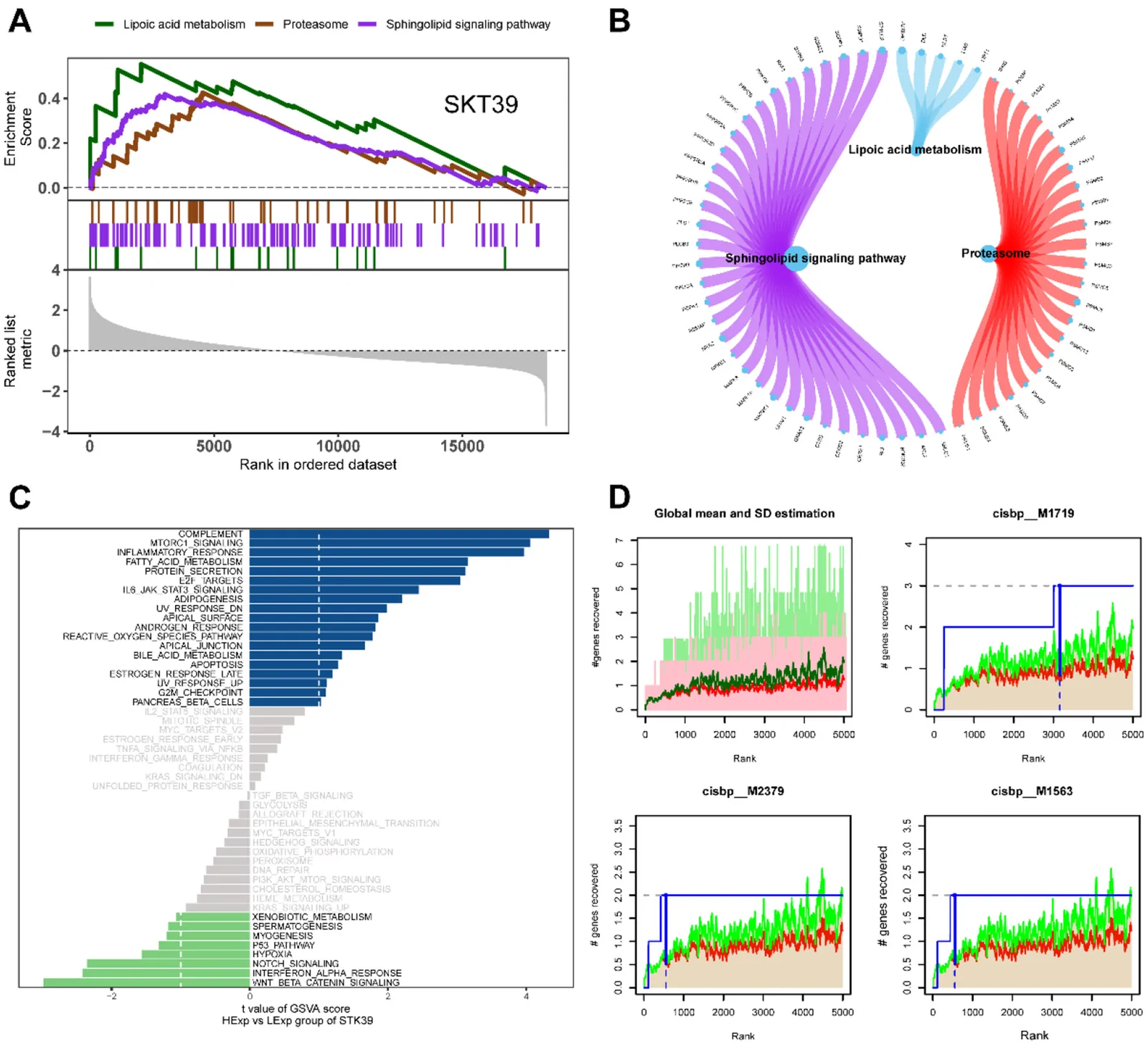
Functional enrichment analysis of STK39 and transcription factor enrichment analysis. **(A)** GSEA of STK39 depicts that the pathways enriched by STK39 include lipoic acid metabolism, the Proteasome, and Sphingolipid signaling. **(B)** Genes associated with the three pathways enriched by STK39 (Purple indicates genes related to the sphingolipid signaling pathway, blue indicates genes related to lipoic acid metabolism, and red indicates genes related to the proteasome). **(C)** GSVA of STK39 depicts that STK39 is enriched in MTORC1 signaling, reactive oxygen species pathway, protein secretion, and other signaling pathways. **(D)** The enriched motifs and corresponding transcription factors of the key genes.

GSVA results showed that high STK39 expression was enriched in mtorc1 signaling, reactive oxygen species pathway, and protein secretion (Figure 9C). In addition, highly expressed NEO1 was enriched in bile acid metabolism, protein secretion, and mtorc1 signaling (Supplementary Figure S5A). PKNOX1 was enriched in KRAS proto-oncogene, GTPase (historically, “Kirsten rat sarcoma viral oncogene homolog”) (KRAS) signaling, IL6, JAK STAT3 signaling, and mtorc1 signaling (Supplementary Figure S5B). High GOLT1B expression was enriched in protein secretion, mtorc1 signaling, IL6, and JAK STAT3 signaling (Supplementary Figure S5C). The highly expressed MTMR12 was enriched in bile acid metabolism, IL6, JAK STAT3 signaling, and MTORC1 signaling (Supplementary Figure S5D).

### Analysis of transcriptional regulation of key genes and their relationship with MMD pathogenic genes

Motif annotation and selection analysis of important genes demonstrated that cisbp _ M1719 had the highest standardized enrichment score (NES: 7.03). The motifs and corresponding key gene transcription factors were analyzed and demonstrated. MMD related genes were obtained from GeneCards (https://www.genecards.org/). von Willebrand factor (VWF), Galactosidase alpha (alpha-galactosidase A) (GLA), SBF2, VCP, NOS3, and IL1B expression levels differed significantly between the two subtypes (Figure 10A). In addition, the expression levels of key genes were potentially related to MMD-related genes. MTMR12 was significantly positively correlated with SBF2 (*r* = 0.882) and STK39 was significantly negatively correlated with VCP (*r* = −0.833) (Figure 10B). No statistically significant correlation was detected between STK39 and RNF213 transcript levels in the analyzed samples (Figure 10B).

**Figure 10:**
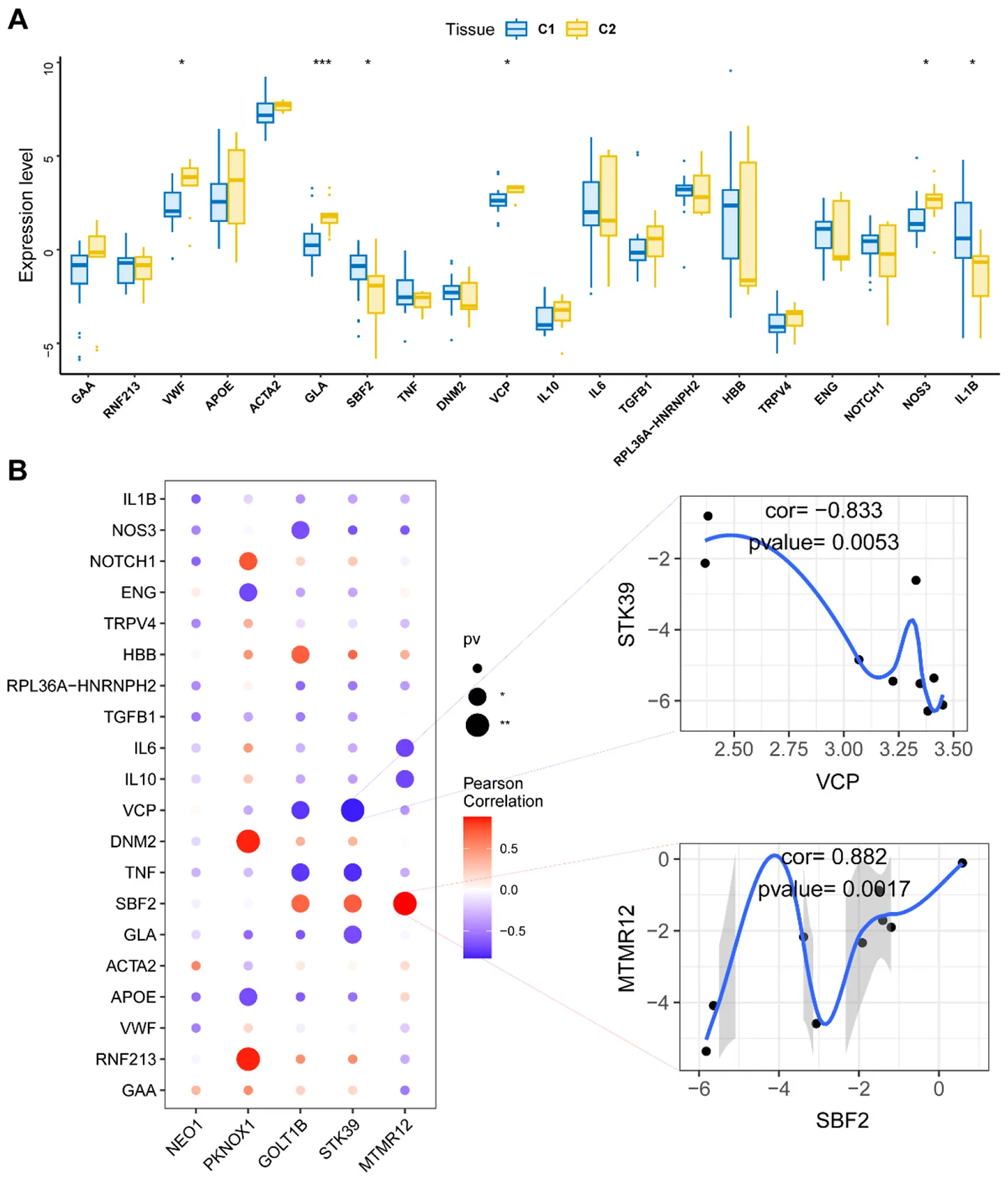
Association of moyamoya disease genes with acetylation subtypes and key genes. **(A)** Analysis of differential expression of Moyamoya disease genes between the two acetylated isoforms (Tissue: blue indicates C1; C2 is shown in yellow. Ordinate: gene expression levels. Abstract: Gene name for moyamoya disease). **(B)** Correlation between the expression levels of the five key genes and the moyamoya disease genes, STK39 shows a significant negative correlation with VCP. MEMR12 also demonstrates a significant positive correlation with SBF2 levels (Ordinate: name of the moyamoya disease gene. Abstract: Five key genes were identified. Red indicates a positive correlation, blue indicates a negative correlation, darker colors indicate stronger correlation, and larger bubbles indicate more statistical significance, *P < 0.05, **P < 0.01).

## Discussion

MMD is a rare and occlusive cerebrovascular disease that is difficult to prevent and treat as its mechanisms are poorly understood (Gonzalez et al., 2023). Herein, we used three public databases for differential analysis and selected five key genes. Relevant immune cell infiltration and functional enrichment analyses were performed. The expression clusters defined using acetylation-regulatory genes were associated with differences in molecular pathways and inferred immune-cell composition. These associations provide hypotheses for further investigation but do not establish an acetylation-driven mechanism in MMD.

Acetylation-related genes are closely related to the acetylation modification of proteins, so they have been widely studied in vascular diseases. Histone-3 acetylation increases Vascular endothelial growth factor (VEGF) expression in human retinal endothelial cells under hypoxic conditions (Leng et al., 2023). In MMD under similar conditions, VEGF was also highly expressed, which may be related to the abnormal expression of acetylation-related genes (Wang et al., 2021). Increased endothelial cells proliferation can promote vascular occlusion and angiogenesis in the ischemic brain tissue of mice (Chen et al., 2020). The pro-growth effect of VEGF may be closely related to vascular endothelial thickening and vascular network formation in the hypoxic and ischemic conditions of MMD. TH-6, a novel tumor-growth inhibitor, was recently reported to inhibit microtubule polymerization and histone deacetylation. Owing to its characteristics, it can cause the internal and peripheral blood vessels of the tumor to lose their normal structure and inhibit tumor growth (Zhu et al., 2022). This also reflects the important role of acetylation in vascular structures regulation. Microtubules are important cytoskeleton components that are involved in diverse vascular cell functions (MacTaggart and Kashina, 2021; Veloso et al., 2024). Although the type of disease is different, MMD also causes abnormal vascular hyperplasia. Therefore, the pathological process of abnormal blood vessels induced by acetylation-related genes in the above-mentioned tumors may also occur in MMD. Thus, the abnormal expression of acetylation-related genes in MMD may indicate vascular pathology.

The five prioritized genes showed lower expression in MMD than in non-MMD surgical controls at the nominal *P-value* level, but none met the transcriptome-wide FDR threshold in the primary reanalysis. STK39 met the within-comparison FDR threshold when MMD samples were compared with epilepsy controls. This additional finding provides an exploratory signal supporting further investigation of STK39, rather than establishing a robust association across control groups. Accordingly, STK39 was retained as an exploratory candidate. Candidate-gene expression was associated with differences in pathway enrichment and inferred immune-cell composition. The possible mechanisms of action of key genes in the pathogenesis of moyamoya disease will be discussed below.

Serine/threonine kinase-39 (STK39, SPAK), a kinase closely related to vascular diseases, a PAPA box (proline and alanine repeats), a nuclear localization signal peptide and a catalytic domain (Johnston et al., 2000). STK39 encodes STE20-related proline/alanine-rich kinase (SPAK), a downstream effector of WNK kinases that regulates cation–chloride cotransport and cellular ion homeostasis (Moriguchi et al., 2005). Genetic studies have implicated the STK39 locus in susceptibility to Parkinson's disease (International Parkinson Disease Genomics Consortium et al., 2011), while a family-based association study identified STK39 as a candidate susceptibility gene for autism (Ramoz et al., 2008). These findings suggest potential neurological relevance, although they do not establish the causal mechanisms underlying these associations. Beyond these genetic links, STK39 has been associated with blood pressure regulation and hypertension, potentially through effects on renal sodium handling (Wang et al., 2009). Experimental studies also support a role in vascular function. In human umbilical vein endothelial cells, SPAK contributes to WNK1-dependent angiogenic responses by supporting endothelial proliferation (Dbouk et al., 2014). In endotoxemic mice, SPAK deficiency reduces excessive nitric oxide production and improves vascular responsiveness to vasoconstrictors, indicating its involvement in vascular dysfunction under inflammatory conditions (Shih et al., 2017). Together, these findings provide a rationale for investigating STK39 in endothelial responses relevant to moyamoya disease (MMD). However, whether and how STK39 contributes to hypoxia-associated endothelial dysfunction in MMD remains to be clarified. Also STK39 may affect blood pressure regulation by regulating reabsorption of water and ions in the kidneys (Mercado et al., 2020). Additionally, STK39 affected NO secretion and vascular reactivity in endotoxin-treated mice (Shih et al., 2017). However, few studies have investigated the role of STK39 in MMD. Herein, STK39 was found to be downregulated in MMD, and enrichment analysis demonstrated that it was significantly correlated with apoptosis and IL6, JAK STAT3, P53 signaling pathway and WNT BETA signaling pathways. Among the MMD pathogenic genes, STK39 was significantly and negatively correlated with transitional endoplasmic reticulum ATPase (VCP).

NEO1 is a member of the Deleted in colorectal carcinoma (DCC) transmembrane protein family and is a receptor/ co-receptor of multiple ligands (Rajagopalan et al., 2004; Wilson and Key, 2006; Zhou et al., 2010). It exerts antiapoptotic effects on vascular endothelial progenitor cells under hypoxic conditions (Jiang et al., 2021). In the ischemic and hypoxic environments of MMD, the above phenomena may occur. However, a reduction in neo1 may diminish this effect, which may cause abnormalities in the number of endothelial progenitor cells. In MMD, the abnormal number of endothelial progenitor cells is one of the initiating factors for the remodeling of pathological arteries (Yu et al., 2020). Therefore, decreased neo1 expression in MMD may promote vascular remodeling via an abnormal number of endothelial progenitor cells.

PBX-regulating protein-1 (Prep1 and Pknox1) is a transcription factor belonging to the family of three-amino acid loop extension homeodomain proteins, which are involved in ontogenesis, metabolism, and tumorigenesis (Micali et al., 2010, 2023; Fernandez-Diaz et al., 2010). Previous studies have indicated that Pknox1 exerts anti-apoptotic effects on muscle cells (Cimmino et al., 2021). The HIF-1 signaling pathway is activated under hypoxia and functions in neuronal survival, neuroinflammation, angiogenesis, glucose metabolism, and blood–brain barrier (Dewhirst, 2009; He et al., 2021). In ischemic stroke, the spatiotemporal expression of HIF-1 can reflect the changes of the stroke. In this study, there was a significant association between Pknox1 and the HIF-1 signaling pathway. This may be a pathological process mediated by the hypoxic environment caused by MMD. Therefore, low Pknox1 levels in MMD may contribute to its pathogenesis by increasing apoptosis of vascular smooth muscle cells.

Golgi transport protein 1 B (GOLT1B), a member of the Golgi family, plays crucial roles in protein transport, processing, and sorting (Tian et al., 2023). Myotubularin-related protein 12 (MTMR12), a member of the MTMR family, has been shown to interact with myotubulin to form oligomers in K562 and Cos7 cells. The MTMRs family is one of the largest and most conserved subfamilies of eukaryotic proteolipid phosphatases (Cox et al., 2013). In addition, MTMR12 is closely related to immune cells.

GSVA and GSEA enrichment analysis demonstrated that STK39, PREP1, GOLT1B, and MTMR12 were negatively correlated with the p53 signaling pathway, and STK39, GOLT1B, and MTMR12 were negatively correlated with the WNT signaling pathway. This suggests that STK39 is involved in the pathological process of vascular occlusion in MMD by affecting the abnormal proliferation of VSMC and Endothelial cells (EC). Accumulating evidence suggests that signal transducers and activators of transcription (STATs), predominantly STAT3, play important roles in angiogenesis in addition to cell survival, proliferation, differentiation, and tumorigenesis under physiological and pathological conditions (Chen and Han, 2007). Additionally, it has been demonstrated that macrophages can regulate the phenotype and apoptosis of VSMC through the STAT3-related signaling pathway (Xue et al., 2022). Additionally, macrophages, an upstream factor of STAT3, showed a significant negative correlation with NEO1 expression in immune infiltration analysis. Moreover, for C1, M0, and M2, macrophages of C2 increased significantly. In vascular inflammation, monocytes migrate to the inflammatory region and differentiate into macrophages due to the induction of chemokines, thereby participating in the pathological process (Jaipersad et al., 2014; Narasimhan et al., 2019). There may be a complex immune-related inflammation in MMD (Asselman et al., 2022). In this study, monocytes exhibited significant differences in different subtypes. This indicates that monocytes and macrophages are among the important drivers of vascular inflammation in MMD. In addition to macrophages, several other immune factors are involved in MMD pathogenesis. Both MTMR12 and PKNOX1 significantly and positively correlated with neutrophil levels. PKNOX1, NEO1, and MTMR12 positively correlated with activated NK cells. Although not significantly correlated with resting NK cells, all three key genes were negatively correlated with resting NK cells. Previous studies have also found abnormal increases and decreases in B, CD4, and NK cells in the peripheral blood of MMD patients (Cai et al., 2019). This suggests that NK cells may be involved in the pathological process of MMD as downstream immune factors of PKNOX1, NEO1, and MTMR12. The inferred immune differences were broadly consistent with a recent single-cell transcriptomic study of superficial temporal arteries in MMD, which demonstrated enhanced immune activity, particularly involving natural killer T cells, and identified functional interactions between immune cells, endothelial cells, and smooth-muscle cells. CIBERSORT estimates relative immune-cell composition using a predefined reference signature, whereas single-cell RNA sequencing resolves cell populations directly. The present deconvolution results should therefore be regarded as supportive and hypothesis-generating rather than as direct validation of specific immune-cell alterations (He et al., 2025c,d).

Overall, the analyses identified candidate genes associated with expression clusters defined by acetylation-regulatory genes and with inferred immune differences in MMD. Functional experiments supported a potential role for STK39 in endothelial responses to hypoxia, while its relationship with acetylation requires further investigation.

## Limitations

The transcriptomic analysis was limited by the small sample size, heterogeneous surgical controls and demographic differences between groups. None of the five prioritized candidates met the transcriptome-wide FDR threshold in the primary reanalysis. Although STK39 and NEO1 met the within-comparison FDR threshold in the exploratory comparison with epilepsy controls, neither met this threshold in the comparison with aneurysm controls. These comparisons reused the discovery samples and do not constitute independent validation. The candidates should therefore be interpreted as hypothesis-generating findings requiring further validation in larger, independent cohorts with appropriately matched controls. The C1 and C2 clusters were defined from the expression of acetylation-related genes rather than direct measurements of histone or non-histone protein acetylation. Therefore, they should not be interpreted as confirmed high- and low-acetylation states. In addition, the pathway and immune-infiltration differences observed between the clusters represent associations and do not establish that acetylation dysregulation causally drives vascular remodeling or inflammation. Independent cohorts and functional studies are required to validate the biological stability and mechanistic significance of these clusters. Hypoxia was selected as a disease-relevant stress condition because progressive arterial stenosis in MMD can result in chronic cerebral hypoperfusion and recurrent ischemic stress. Nevertheless, the present *in vitro* model represents an acute and simplified reduction in oxygen availability. It does not reproduce the chronic or fluctuating hemodynamic environment, altered shear stress, interactions among endothelial cells, smooth-muscle cells and immune cells, or other components of MMD vascular pathology.

## Conclusions

In conclusion, this study identified acetylation-associated gene-expression patterns that may reflect molecular heterogeneity in MMD and prioritized STK39 as a candidate gene associated with vascular dysfunction. Functional experiments further indicated that STK39 may influence endothelial responses to hypoxic stress. However, the present findings do not establish acetylation-dependent regulation of STK39, and the potential relationship between acetylation and STK39 requires further mechanistic investigation.

## Data Availability

The original contributions presented in the study are included in the article/Supplementary material, further inquiries can be directed to the corresponding authors.
