# Supplementary material for: STK39 is associated with endothelial dysfunction and inflammatory activation under hypoxic conditions in moyamoya disease
Source: Front Hum Neurosci. 2026 Oct 14;20:1854099. doi: 10.3389/fnhum.2026.1854099 (PMC13713786; doi:10.3389/fnhum.2026.1854099)
Supplement: Supplementary file 1 [file Data_Sheet_1.pdf]

Figure S1:

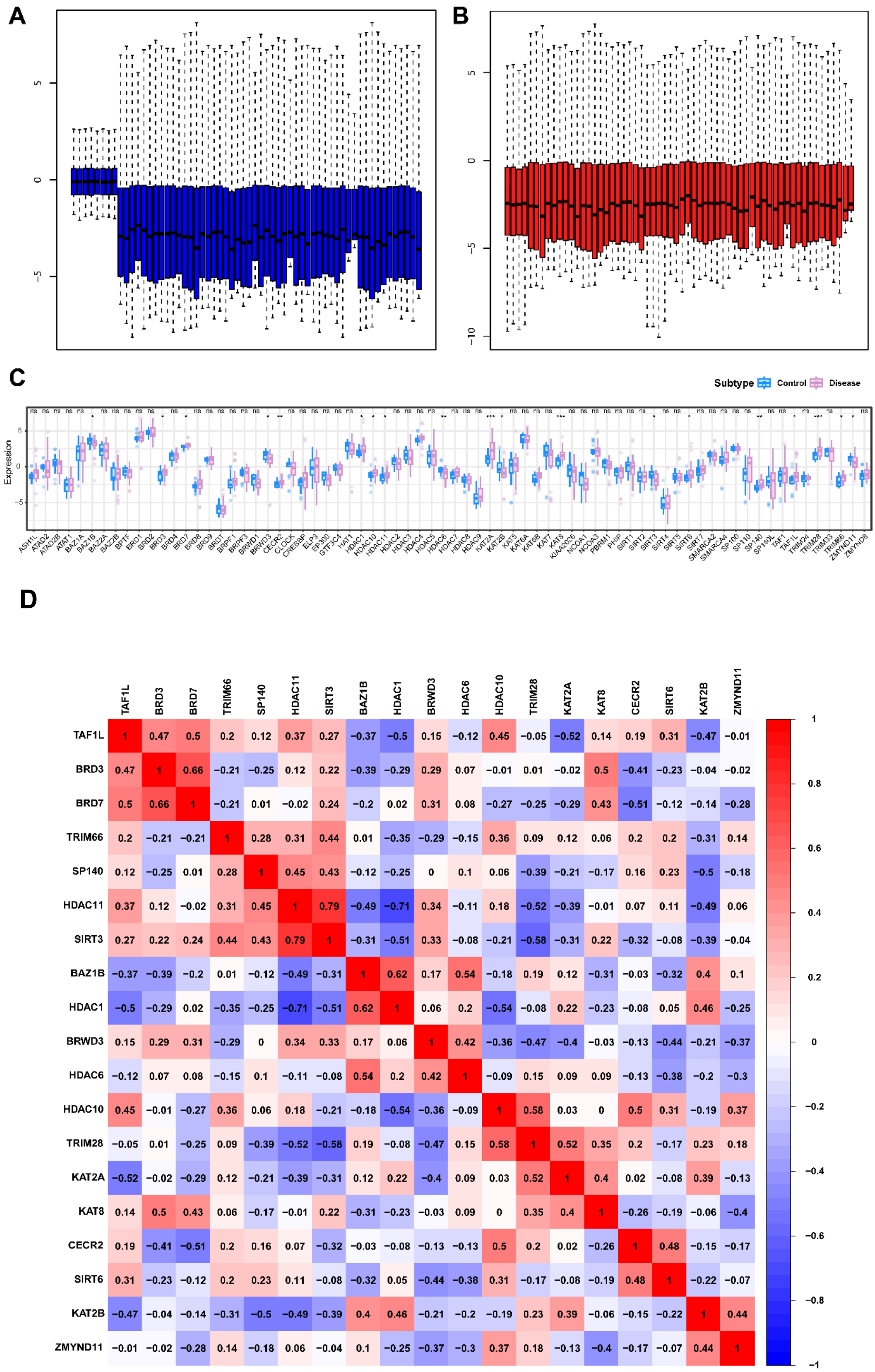
A
B
C
Subtype Control Disease
Expression
D
TAF1L BRD3 BRD7 TRIM66 SP140 HDAC11 SIRT3 BAZ1B HDAC1 BRWD3 HDAC6 HDAC10 TRIM28 KAT2A KAT8 CECR2 SIRT6 KAT2B ZMYND11
TAF1L 1 0.47 0.5 0.2 0.12 0.37 0.27 −0.37 −0.5 0.15 −0.12 0.45 −0.05 −0.52 0.14 0.19 0.31 −0.47 −0.01
BRD3 0.47 1 0.66 −0.21 −0.25 0.12 0.22 −0.39 −0.29 0.29 0.07 −0.01 0.01 −0.02 0.5 −0.41 −0.23 −0.04 −0.02
BRD7 0.5 0.66 1 −0.21 0.01 −0.02 0.24 −0.2 0.02 0.31 0.08 −0.27 −0.25 −0.29 0.43 −0.51 −0.12 −0.14 −0.28
TRIM66 0.2 −0.21 −0.21 1 0.28 0.31 0.44 0.01 −0.35 −0.29 −0.15 0.36 0.09 0.12 0.06 0.2 0.2 −0.31 0.14
SP140 0.12 −0.25 0.01 0.28 1 0.45 0.43 −0.12 −0.25 0 0.1 0.06 −0.39 −0.21 −0.17 0.16 0.23 −0.5 −0.18
HDAC11 0.37 0.12 −0.02 0.31 0.45 1 0.79 −0.49 −0.71 0.34 −0.11 0.18 −0.52 −0.39 −0.01 0.07 0.11 −0.49 0.06
SIRT3 0.27 0.22 0.24 0.44 0.43 0.79 1 −0.31 −0.51 0.33 −0.08 −0.21 −0.58 −0.31 0.22 −0.32 −0.08 −0.39 −0.04
BAZ1B −0.37 −0.39 −0.2 0.01 −0.12 −0.49 −0.31 1 0.62 0.17 0.54 −0.18 0.19 0.12 −0.31 −0.03 −0.32 0.4 0.1
HDAC1 −0.5 −0.29 0.02 −0.35 −0.25 −0.71 −0.51 0.62 1 0.06 0.2 −0.54 −0.08 0.22 −0.23 −0.08 0.05 0.46 −0.25
BRWD3 0.15 0.29 0.31 −0.29 0 0.34 0.33 0.17 0.06 1 0.42 −0.36 −0.47 −0.4 −0.03 −0.13 −0.44 −0.21 −0.37
HDAC6 −0.12 0.07 0.08 −0.15 0.1 −0.11 −0.08 0.54 0.2 0.42 1 −0.09 0.15 0.09 0.09 −0.13 −0.38 −0.2 −0.3
HDAC10 0.45 −0.01 −0.27 0.36 0.06 0.18 −0.21 −0.18 −0.54 −0.36 −0.09 1 0.58 0.03 0 0.5 0.31 −0.19 0.37
TRIM28 −0.05 0.01 −0.25 0.09 −0.39 −0.52 −0.58 0.19 −0.08 −0.47 0.15 0.58 1 0.52 0.35 0.2 −0.17 0.23 0.18
KAT2A −0.52 −0.02 −0.29 0.12 −0.21 −0.39 −0.31 0.12 0.22 −0.4 0.09 0.03 0.52 1 0.4 0.02 −0.08 0.39 −0.13
KAT8 0.14 0.5 0.43 0.06 −0.17 −0.01 0.22 −0.31 −0.23 −0.03 0.09 0 0.35 0.4 1 −0.26 −0.19 −0.06 −0.4
CECR2 0.19 −0.41 −0.51 0.2 0.16 0.07 −0.32 −0.03 −0.08 −0.13 −0.13 0.5 0.2 0.02 −0.26 1 0.48 −0.15 −0.17
SIRT6 0.31 −0.23 −0.12 0.2 0.23 0.11 −0.08 −0.32 0.05 −0.44 −0.38 0.31 −0.17 −0.08 −0.19 0.48 1 −0.22 −0.07
KAT2B −0.47 −0.04 −0.14 −0.31 −0.5 −0.49 −0.39 0.4 0.46 −0.21 −0.2 −0.19 0.23 0.39 −0.06 −0.15 −0.22 1 0.44
ZMYND11 −0.01 −0.02 −0.28 0.14 −0.18 0.06 −0.04 0.1 −0.25 −0.37 −0.3 0.37 0.18 −0.13 −0.4 −0.17 −0.07 0.44 1

Inclusion and differential analysis of Moyamoya disease and acetylation-related genes data (plus diverse numbers of people)

A: Sample-wise distributions of normalized log2 gene-expression values before batch adjustment. Each box represents one individual GEO expression profile.

B: Corresponding expression distributions after batch adjustment. Principal component analysis before and after adjustment is provided to demonstrate changes in dataset-associated technical variation. C: Analysis of differences in acetylation-related genes between the control and disease groups. (Blue: control group; Pink: disease group; ordinate: gene expression level, abscissa: intersection of acetylation-related genes between control group and disease group; ns represents no statistical significance, P < 0.05, ** P < 0.01, *** P < 0.001). D:Correlation analysis of acetylated genes. (Red indicates a positive correlation, blue

indicates negative correlation, and darker colors indicate a deeper correlation.)

Figure S2:

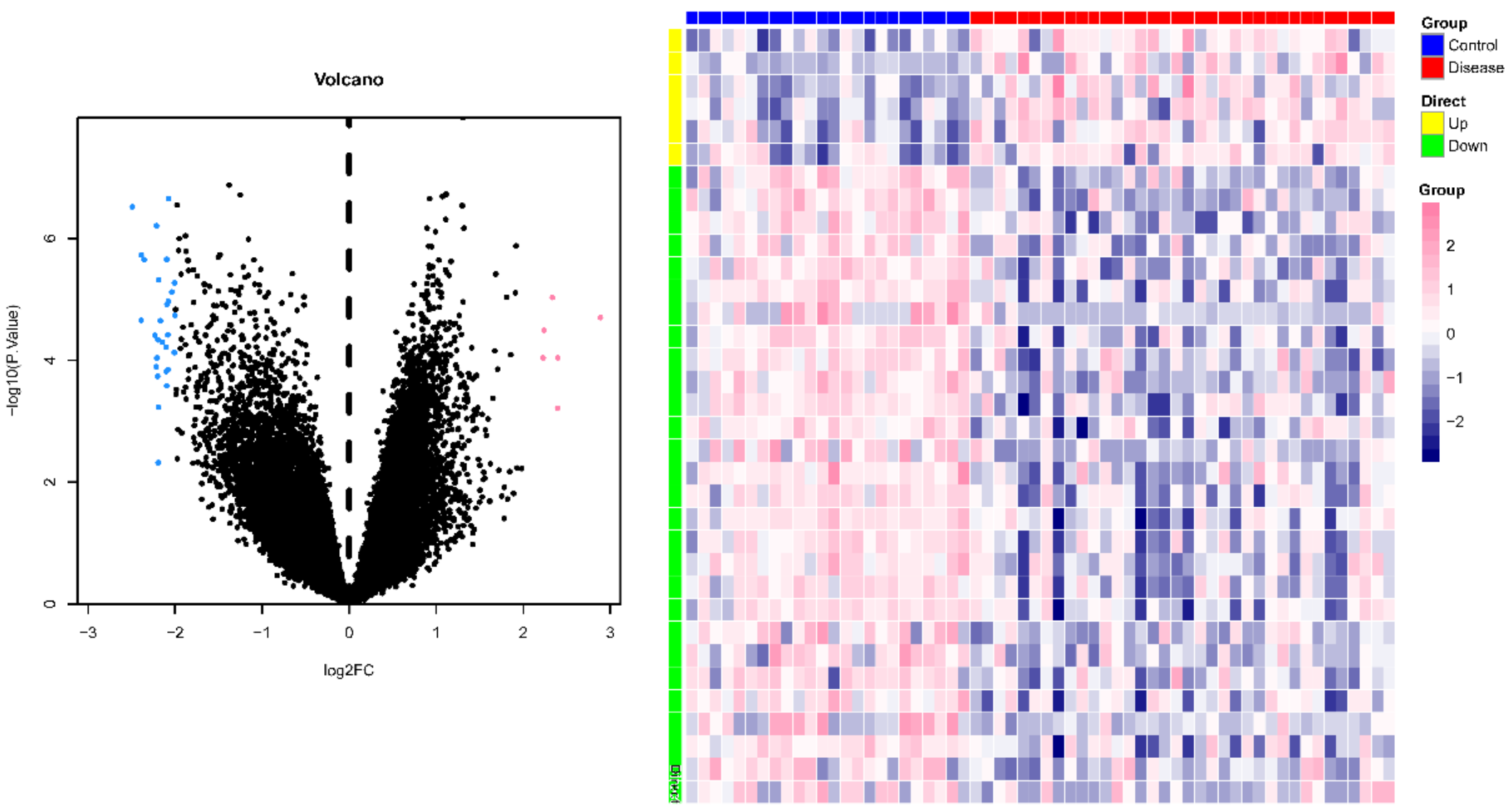

Differential gene analysis between the control and disease groups. Cut genes (volcano plot: blue, red upregulated genes, filter condition: P. V value < 0.05, and | logFC | > 2, a total of 34 selected differentially expressed genes ,Heat map:
abscissa; blue, control group; red, disease group. Ordinate: yellow indicates upregulated genes screened in the volcano plot, and green indicates downregulated genes screened in the volcano plot. Red represents upregulated gene expression levels and darker colors represent upregulated genes. Blue represents the downregulation of

gene expression levels, and darker colors represent more downregulation) Transcriptome-wide differential expression analysis between the MMD and non-MMD control groups. The volcano plot and heatmap show the 34 differentially expressed genes identified using P < 0.05 and |log2 fold change| > 2, including six upregulated and 28 downregulated genes.

Figure S3:

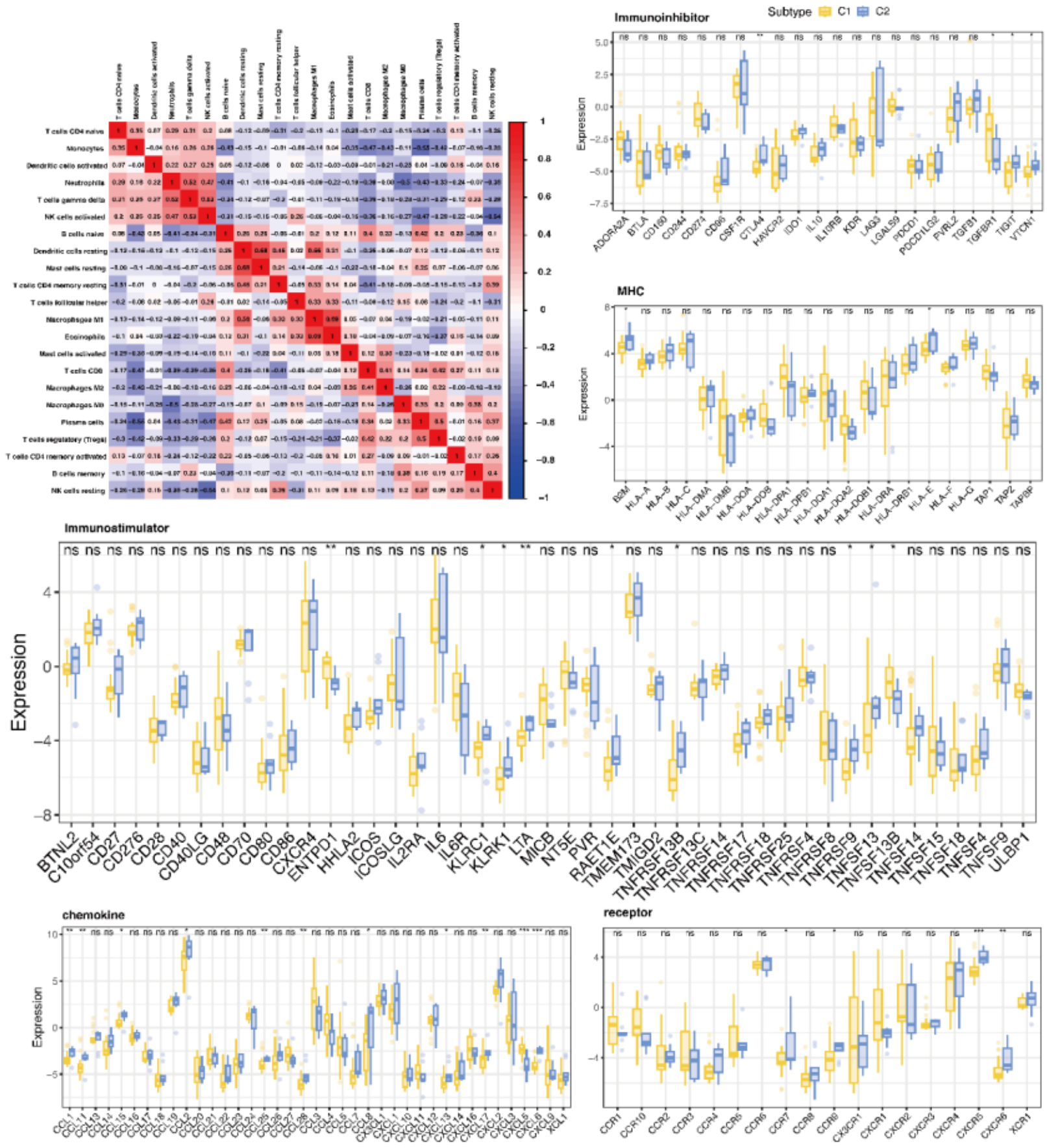

Correlation and difference analyses of multiple immune factors in moyamoya disease. Heat map: Interaction between different types of immune cells in moyamoya disease (horizontal and vertical coordinates: immune cell name; red indicates a positive correlation, blue indicates a negative correlation, and darker colors indicate stronger correlations). Box plot: The top left side identifies the type of immune factor, ordinate represents the expression level of the factor, and abscissa represents the name of each factor type. (ns, no statistical significance; P < 0.05; ** P < 0.01; *** P < 0.001)

Figure S4:

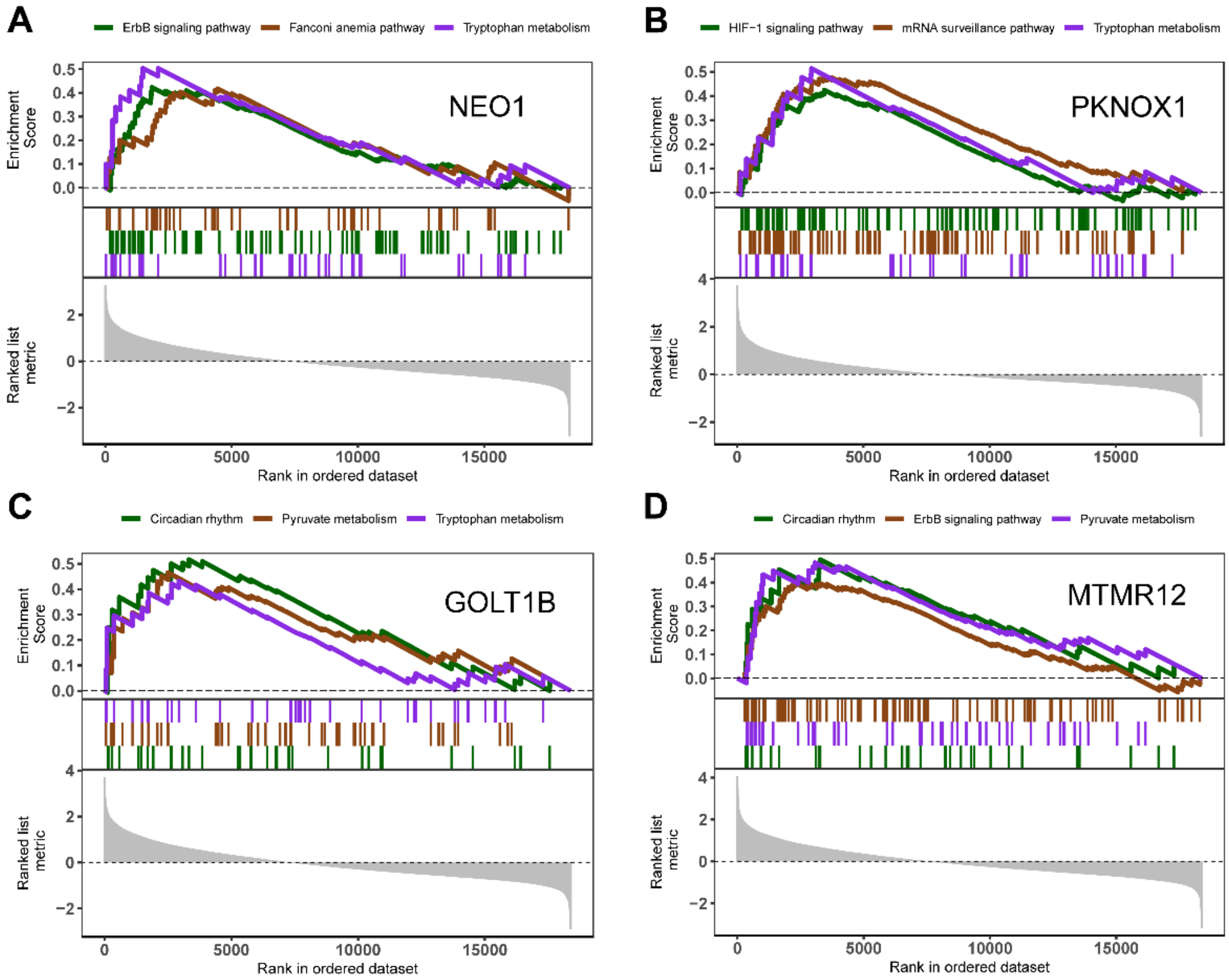

Sup 4： GSEA of NEO1, PKNOX1, GOLT1B, and MTMR12

A: GSEA of NEO1 depicts that the pathways enriched by NEO1 are the ErbB signaling pathway, Fanconi anemia pathway, and tryptophan metabolism.

B： GSEA of PKNOX1 depicts that the pathway enriched by PKNOX1 are HIF−1

signaling pathway, mRNA surveillance pathway, Tryptophan metabolism.
C: GSEA of GOLT1B depicts that the pathways enriched by GOLT1B are Circadian rhythm, pyruvate metabolism, and tryptophan metabolism.
D: GSEA of MTMR12 depicts that the pathways enriched by MTMR12 include circadian
rhythm, ErbB signaling pathway, and pyruvate metabolism.

Figure S5:

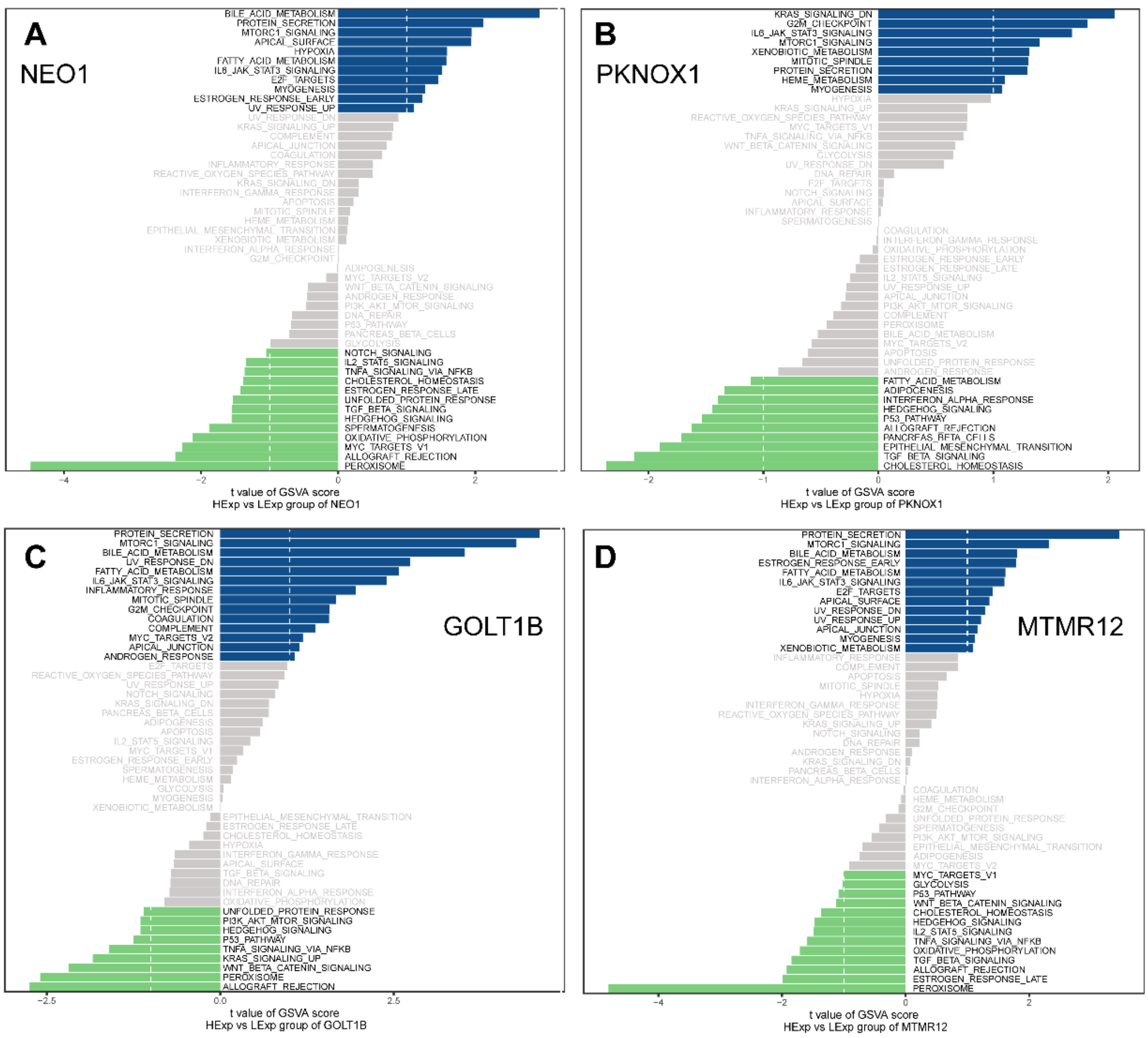

Sup 5：GSVA of high and low expression of NEO1, PKNOX1, GOLT1B, and MTMR12

A: GSVA of NEO1 shows that the pathways enriched by NEO1 are BILE ACID METABOLISM, PROTEIN SECRETION、MTORC1 SIGNALING and etc.

B: GSVA of PKNOX1 depicts that the pathways enriched by PKNOX1 are KRAS SIGNALING_DN, IL6 JAK STAT3 SIGNALING、MTORC1 SIGNALING and etc.

C: GSVA of GOLT1B depicts that the pathways enriched by GOLT1B are PROTEIN SECRETION, MTORC1 SIGNALING, and IL6 JAK STAT3 SIGNALING.

D: GSVA of MTMR12 shows that the pathways enriched by MTMR12 are BILE ACID METABOLISM, IL6 JAK STAT3 SIGNALING、MTORC1 SIGNALING and etc.

Table S1: Detailed clinical information for the participants.

Profile ID	disease	Age(year)	sex	Taken medicines	Antibiotics for the operation	Clinical presentation	Duration until surgery from the last clinical presentation	Subtype of MMD	Aneurysm location/size	Epileptic origin
1	MMD	64	F	Cilostazol 100 mg/day, atorvastatin 10 mg/day	Cefazolin 1 g	ICH	2 months	One side	NR	NR
2	MMD	47	F	Aspirin 100 mg/day, Lansoprazole 15 mg/day, Atorvastatin Calcium Hydrate 10 mg/day, Tsumura Goreisan 2.5g/day, Bepotastine Besilate 10 mg/day	Cefazolin 1 g	IF	8 months	Bilateral	NR	NR
3	MMD	48	F	Amlodipine Besilate 5 mg/day	Cefazolin 1 g	ICH	6 months	Bilateral	NR	NR
4	MMD	49	F	Aspirin 100 mg/day	Cefazolin 1 g	TIA	9 months	Bilateral	NR	NR
5	MMD	59	F	Cilostazol 100 mg/day, Esomeprazole Magnesium Hydrate 20 mg/day	Cefazolin 1 g	IF	11 month	Bilateral	NR	NR
6	MMD	50	F	Cilostazol 100 mg/day, Olopatadine Hydrochloride 5 mg/day	Cefazolin 1 g	TIA	1 month	Bilateral	NR	NR
7	MMD	45	F	Aspirin 100 mg/day, Lansoprazole 15 mg/day, Clopidogrel Sulfate 75 mg/day, Pitavastatin Calcium Hydrate 2 mg/day	Cefazolin 1 g	IF	1 year and 1 month	Bilateral	NR	NR

Profile ID	disease	Age(year)	sex	Taken medicines	Antibiotics for the operation	Clinical presentation	Duration until surgery from the last clinical presentation	Subtype of MMD	Aneurysm location/size	Epileptic origin
8	MMD	48	F	Aspirin 100 mg/day, Lansoprazole 15 mg/day, Celecoxib 100 mg/day	Cefazolin 1g	TIA	3 months	Bilateral	NR	NR
9	MMD	51	M	Aspirin 100 mg, Etizolam 0.5 mg/day, Olmesartan Medoxomil 10 mg/day, Azelnidipine 8 mg/day	Cefazolin 1 g	TIA	1 month	Bilateral	NR	NR
10	MMD	53	F	Aspirin 100 mg/day, Lansoprazole 15 mg/day	Cefazolin 1 g	TIA	7 months	Bilateral	NR	NR
11	MMD	43	F	Aspirin 100 mg, /day Lansoprazole 15 mg/day, Telmisartan 40 mg/day	Cefazolin 1 g	TIA	1 year and 2 months	Bilateral	NR	NR
12	MMD	34	F	Aspirin 100 mg/day	Cefazolin 1 g	TIA	5 months	Bilateral	NR	NR
13	MMD	29	F	Levetiracetam 1000 mg/day, Phenytoin 300 mg/day, Sodium Ferrous Citrate 100 mg/day	Cefazolin 1 g	ICH	7 months	Bilateral	NR	NR
14	MMD	32	M	Aspirin 100 mg/day, Lansoprazole 15 mg/day	Cefazolin 1 g	TIA	Several days	Bilateral	NR	NR
15	MMD	7	F	NR	Cefazolin 1 g	TIA	Several days	Bilateral	NR	NR
16	MMD	27	M	Aspirin 100 mg/day, Esomeprazole Magnesium Hydrate 20 mg/day	Cefazolin 1 g	TIA	8 months	Bilateral	NR	NR

Profile ID	disease	Age(year)	sex	Taken medicines	Antibiotics for the operation	Clinical presentation	Duration until surgery from the last clinical presentation	Subtype of MMD	Aneurysm location/size	Epileptic origin
17	MMD	5	M	Aspirin 30 mg/day	Cefazolin 0.3 g	IF	3 months	Bilateral	NR	NR
18	MMD	39	M	NR	Cefazolin 1 g	TIA	7 months	Bilateral	NR	NR
19	MMD	11	M	Cilostazol 150 mg/day	Cefazolin 0.5 g	TIA	2 months	Bilateral	NR	NR
20	MMD	46	F	Sodium Ferrous Citrate 50 mg/day, Olopatadine Hydrochloride 10 mg/day	Cefazolin 1 g	TIA	2 months	Bilateral	NR	NR
21	MMD	35	F	Pregabalin 225 mg/day, Amitriptyline Hydrochloride 35 mg/day, Desloratadine 5 mg/day	Cefazolin 1 g	TIA	8 months	Bilateral	NR	NR
22	MMD	48	F	Aspirin 100 mg/day, Lansoprazole 15 mg/day, Celecoxib 100 mg/day	Cefazolin 1 g	TIA	3 months	Bilateral	NR	NR
23	MMD	51	M	Aspirin 100 mg, Etizolam 0.5 mg/day, Olmesartan Medoxomil 10 mg/day, Azelnidipine 8 mg/day	Cefazolin 1 g	TIA	1 month	Bilateral	NR	NR
24	MMD	53	F	Aspirin 100 mg/day, Lansoprazole 15 mg/day	Cefazolin 1 g	TIA	7 months	Bilateral	NR	NR
25	MMD	43	F	Aspirin 100 mg, /day Lansoprazole 15 mg/day, Telmisartan 40 mg/day	Cefazolin 1 g	TIA	14 months	Bilateral	NR	NR

Profile ID	disease	Age(year)	sex	Taken medicines	Antibiotics for the operation	Clinical presentation	Duration until surgery from the last clinical presentation	Subtype of MMD	Aneurysm location/size	Epileptic origin
26	IA	62	F	Loxoprofen Sodium Hydrate 180 mg/day, Rebamipide 300 mg/day, Brotizolam 0.25 mg/day, Diazepam 2 mg/day	Cefazolin 1 g	NR	NR	NR	ICA/27mm	NR
27	IA	78	F	Etizolam 0.5 mg/day, Tandospirone Citrate 20 mg/day, Kallidinogenase 100 units/day, Magnesium Oxide 660 mg/day	Cefazolin 1 g	NR	NR	NR	Cavernous/28mm	NR
28	IA	79	F	Aspirin 100 mg/day, Cilostazol 100 mg/day, Acetaminophen 1200 mg/day, Mecobalamin 1500 µg/day, Benidipine Hydrochloride 4 mg/day, Ezetimibe 10 mg/day, Rosuvastatin Calcium 5 mg/day, Candesartan Cilexetil 8 mg/day	Cefazolin 1 g	Oculomotor nerve palsy	1 month	NR	Cavernous/26mm	NR
29	IA	70	F	Valsartan 160 mg/day, Amlodipine Besilate 10 mg/day, Imidapril Hydrochloride 5 mg/day, Doxazosin Mesilate 2 mg/day, methotrexate 10 mg/week	Cefazolin 1 g	Oculomotor nerve palsy	Several days	NR	Cavernous/20mm	NR

Profile ID	disease	Age(year)	sex	Taken medicines	Antibiotics for the operation	Clinical presentation	Duration until surgery from the last clinical presentation	Subtype of MMD	Aneurysm location/size	Epileptic origin
30	IA	65	F	Clopidogrel Sulfate 75 mg/day, Atorvastatin Calcium Hydrate 10 mg/day, L-Carbocisteine 1500 mg, Mecobalamin 1500 µg/day, d-Chlorpheniramine Maleate 6 mg/day, Betamethasone 0.5mg/day, d-Chlorpheniramine Maleate 4 mg/day, Olopatadine Hydrochloride 20 mg/day	Cefazolin 1 g	Oculomotor nerve palsy, head ache	Several days	NR	Cavernous/27mm	NR
31	IA	71	F	Aspirin 100 mg/day, Esomeprazole Magnesium Hydrate 20 mg/day, Valsartan 80 mg/day, Amlodipine Besilate 5 mg/day, Suvorexant 15 mg/day	Cefazolin 1 g	NR	NR	NR	ICA/21mm	NR
32	IA	62	F	Loxoprofen Sodium Hydrate 180 mg/day, Rebamipide 300 mg/day, Brotizolam 0.25 mg/day, Diazepam 2 mg/day	Cefazolin 1 g	None	NR	NR	ICA/27mm	NR
33	IA	78	F	Etizolam 0.5 mg/day, Tandospirone Citrate 20 mg/day, Kallidinogenase 100 units/day, Magnesium Oxide 660 mg/day	Cefazolin 1 g	None	NR	NR	Cavernous/28mm	NR

Profile ID	disease	Age(year)	sex	Taken medicines	Antibiotics for the operation	Clinical presentation	Duration until surgery from the last clinical presentation	Subtype of MMD	Aneurysm location/size	Epileptic origin
34	IA	79	F	Aspirin 100 mg/day, Cilostazol 100 mg/day, Acetaminophen 1200 mg/day, Mecobalamin 1500 µg/day, Benidipine Hydrochloride 4 mg/day, Ezetimibe 10 mg/day, Rosuvastatin Calcium 5 mg/day, Candesartan Cilexetil 8 mg/day	Cefazolin 1 g	ONP	1 month	NR	Cavernous/26mm	NR
35	IA	70	F	Valsartan 160 mg/day, Amlodipine Besilate 10 mg/day, Imidapril Hydrochloride 5 mg/day, Doxazosin Mesilate 2 mg/day, methotrexate 10 mg/week	Cefazolin 1 g	ONP	Several days	NR	Cavernous/20mm	NR
36	EPI	56	M	Levetiracetam 2000 mg/day, Lamotrigine 300 mg/day, Perampanel Hydrate 4 mg/day, Pravastatin Sodium 20 mg/day	Cefazolin 1 g	Seizures	Several days	NR	NR	Temporal lobe
37	EPI	14	M	Lacosamide 300 mg/day, Clobazam 15 mg/day, Perampanel Hydrate 6 mg/day	Cefazolin 1 g	Seizures	Several days	NR	NR	Frontal lobe
38	EPI	20	M	Lacosamide 400 mg/day, Lamotrigine 200 mg/day, Carbamazepine 400 mg/day	Cefazolin 1 g	Seizures	Several days	NR	NR	Frontal lobe

Profile ID	disease	Age(year)	sex	Taken medicines	Antibiotics for the operation	Clinical presentation	Duration until surgery from the last clinical presentation	Subtype of MMD	Aneurysm location/size	Epileptic origin
39	EPI	43	F	Carbamazepine 300 mg/day, Atorvastatin Calcium Hydrate 5 mg/day, Fexofenadine Hydrochloride 120 mg/day	Cefazolin 1 g	Seizures	Several days	NR	NR	Temporal lobe
40	EPI	2	M	Levetiracetam 325 mg/day, Zonisamid 16 mg/day	Cefotaxime 0.5 g	Seizures	Several days	NR	NR	Frontal lobe

Abbreviations: MMD, moyamoya disease; F, female; M, male; IA, intracranial aneurysm; EPI, epilepsy; ONP, ocular nerve palsy; ICH, intracerebral hemorrhage; IF, infarction; TIA, transient ischemic attack; ICA, internal carotid artery; MCA, middle cerebral artery; NR, not reported.

Table s2. List of the histone acetylation modulator proteins in the human genome

Name	HGNC Symbol	Protein Groups	Ensembl Gene ID	Genomic Location
**Lysine acetyltransferase 6A**	KAT6A	Writer	ENSG00000083168	8:41929479-42051990
**Lysine acetyltransferase 6B**	KAT6B	Writer	ENSG00000156650	10:74825582-75032622
**Lysine acetyltransferase 7**	KAT7	Writer	ENSG00000136504	17:49788555-49835030
**Lysine acetyltransferase 5**	KAT5	Writer	ENSG00000172977	11:65711996-65719604
**Lysine acetyltransferase 8**	KAT8	Writer	ENSG00000103510	16:31115754-31131393
**Clock circadian regulator**	CLOCK	Writer	ENSG00000134852	4:55427903-55547138
**Histone acetyltransferase 1**	HAT1	Writer	ENSG00000128708	2:171922448-171983682
**Elongator acetyltransferase complex subunit 3**	ELP3	Writer	ENSG00000134014	8:28089673-28191156
**Lysine acetyltransferase 2A**	KAT2A	Writer and Reader	ENSG00000108773	17:42113108-42121358
**Lysine acetyltransferase 2B**	KAT2B	Writer and Reader	ENSG00000114166	3:20040023-20154404
**TATA-box binding protein associated factor 1**	TAF1	Writer and Reader	ENSG00000147133	X:71366239-71532374
**TATA-box binding protein associated factor 1 like**	TAF1L	Writer and Reader	ENSG00000122728	9:32629454-32635669
**Alpha tubulin acetyltransferase 1**	ATAT1	Writer	ENSG00000137343	6:30626842-30646823
**CREB binding protein**	CREBBP	Writer and Reader	ENSG00000005339	16:3725054-3880726

**E1A binding protein p300**	EP300	Writer and Reader	ENSG00000100393	22:41091786-41180079
**General transcription factor IIIC subunit 4**	GTF3C4	Writer	ENSG00000125484	9:132670035-132694955
**Nuclear receptor coactivator 1**	NCOA1	Writer	ENSG00000084676	2:24491914-24770702
**Nuclear receptor coactivator 3**	NCOA3	Writer	ENSG00000124151	20:47501902-47656877
**SP140 nuclear body protein**	SP140	Reader	ENSG00000079263	2:230203110-230313215
**SP140 nuclear body protein like**	SP140L	Reader	ENSG00000185404	2:230327184-230403732
**SP100 nuclear antigen**	SP100	Reader	ENSG00000067066	2:230415942-230544090
**SP110 nuclear body protein**	SP110	Reader	ENSG00000135899	2:230167293-230225729
**Tripartite motif containing 28**	TRIM28	Reader	ENSG00000130726	19:58544091-58550722
**Tripartite motif containing 33**	TRIM33	Reader	ENSG00000197323	1:114392777-114511160
**Tripartite motif containing 24**	TRIM24	Reader	ENSG00000122779	7:138460334-138589993
**Tripartite motif containing 66**	TRIM66	Reader	ENSG00000166436	11:8612037-8682694
**Bromodomain adjacent to zinc finger domain 1A**	BAZ1A	Reader	ENSG00000198604	14:34752731-34875647
**SWI/SNF related, matrix associated, actin dependent regulator of chromatin, subfamily a, member 2**	SMARCA2	Reader	ENSG00000080503	9:1980290-2193624
**SWI/SNF related, matrix associated, actin dependent regulator of chromatin, subfamily a, member 4**	SMARCA4	Reader	ENSG00000127616	19:10960825-11079426
**ASH1 like histone lysine methyltransferase**	ASH1L	Reader	ENSG00000116539	1:155335268-155562807
**Bromodomain adjacent to zinc finger domain 1B**	BAZ1B	Reader	ENSG00000009954	7:73440398-73522278

**Polybromo 1**	PBRM1	Reader	ENSG00000163939	3:52545352-52685917
**Zinc finger MYND-type containing 8**	ZMYND8	Reader	ENSG00000101040	20:47209214-47356889
**Zinc finger MYND-type containing 11**	ZMYND11	Reader	ENSG00000015171	10:134465-254637
**Bromodomain containing 4**	BRD4	Reader	ENSG00000141867	19:15235519-15332545
**Bromodomain PHD finger transcription factor**	BPTF	Reader	ENSG00000171634	17:67825524-67984378
**Bromodomain containing 2**	BRD2	Reader	ENSG00000204256	6:32968660-32981505
**Bromodomain testis associated**	BRDT	Reader	ENSG00000137948	1:91949371-92014426
**Bromodomain containing 3**	BRD3	Reader	ENSG00000169925	9:134030305-134068535
**Lysine methyltransferase 2A**	KMT2A	Reader	ENSG00000118058	11:118436490-118526832
**CECR2, histone acetyl-lysine reader**	CECR2	Reader	ENSG00000099954	22:17359949-17558149
**KIAA2026**	KIAA2026	Reader	ENSG00000183354	9:5881596-6007901
**Bromodomain containing 1**	BRD1	Reader	ENSG00000100425	22:49773283-49827512
**Bromodomain and PHD finger containing 3**	BRPF3	Reader	ENSG00000096070	6:36196744-36232790
**Bromodomain and PHD finger containing 1**	BRPF1	Reader	ENSG00000156983	3:9731729-9748018
**Bromodomain containing 7**	BRD7	Reader	ENSG00000166164	16:50313487-50368934
**Bromodomain containing 9**	BRD9	Reader	ENSG00000028310	5:850291-892824
**Bromodomain adjacent to zinc finger domain 2A**	BAZ2A	Reader	ENSG00000076108	12:56595596-56636816
**Bromodomain adjacent to zinc finger domain 2B**	BAZ2B	Reader	ENSG00000123636	2:159318979-159616692

**ATPase family, AAA domain containing 2**	ATAD2	Reader	ENSG00000156802	8:123319850-123416350
**ATPase family, AAA domain containing 2B**	ATAD2B	Reader	ENSG00000119778	2:23748664-23927114
**Bromodomain containing 8**	BRD8	Reader	ENSG00000112983	5:138139766-138178986
**Bromodomain and WD repeat domain containing 3**	BRWD3	Reader	ENSG00000165288	X:80670854-80809688
**Bromodomain and WD repeat domain containing 1**	BRWD1	Reader	ENSG00000185658	21:39184176-39321559
**Pleckstrin homology domain interacting protein**	PHIP	Reader	ENSG00000146247	6:78935867-79078236
**Histone deacetylase 1**	HDAC1	Eraser	ENSG00000116478	1:32292086-32333635
**Histone deacetylase 2**	HDAC2	Eraser	ENSG00000196591	6:113933028-114011308
**Histone deacetylase 3**	HDAC3	Eraser	ENSG00000171720	5:141620876-141636870
**Histone deacetylase 8**	HDAC8	Eraser	ENSG00000147099	X:72329516-72573103
**Histone deacetylase 11**	HDAC11	Eraser	ENSG00000163517	3:13479724-13506424
**Histone deacetylase 10**	HDAC10	Eraser	ENSG00000100429	22:50245183-50251405
**Histone deacetylase 6**	HDAC6	Eraser	ENSG00000094631	X:48801377-48824982
**Histone deacetylase 7**	HDAC7	Eraser	ENSG00000061273	12:47782722-47833132
**Histone deacetylase 4**	HDAC4	Eraser	ENSG00000068024	2:239048168-239401654
**Histone deacetylase 5**	HDAC5	Eraser	ENSG00000108840	17:44076746-44123702

**Histone deacetylase 9**	HDAC9	Eraser	ENSG00000048052	7:18086949-19002416
**Sirtuin 6**	SIRT6	Eraser	ENSG00000077463	19:4174109-4182604
**Sirtuin 3**	SIRT3	Eraser	ENSG00000142082	11:215458-236931
**Sirtuin 1**	SIRT1	Eraser	ENSG00000096717	10:67884669-67918390
**Sirtuin 2**	SIRT2	Eraser	ENSG00000068903	19:38878555-38899862
**Sirtuin 7**	SIRT7	Eraser	ENSG00000187531	17:81911939-81921323
**Sirtuin 4**	SIRT4	Eraser	ENSG00000089163	12:120302316-120313249
**Sirtuin 5**	SIRT5	Eraser	ENSG00000124523	6:13574529-13615158

This table is derived from previous research.[1]

Table S3. Clinical characteristics and sample information of the patients for Elisa.

**No. of Patients**	**Groups**	**Sex**	**Age (years)**	**Hypertension**	**Diabetes**	**Coronary heart disease**	**Hyperlipidemia**	**Smoking history**	**Alcohol taking**	**Duration of symptoms (months)**	**Suzuki stage**	**Sample**
1	MMD	F	19	No	No	No	No	No	No	4	L1/R1	Serum
2	MMD	F	43	No	No	No	No	No	No	2	L2/R3	Serum
3	MMD	M	17	No	No	No	No	No	No	42	L4/R5	Serum
4	HC	F	29	No	No	No	No	No	No	No	No	Serum
5	HC	M	31	No	No	No	No	No	No	No	No	Serum
6	HC	M	35	No	No	No	No	No	No	No	No	Serum

MMD, moyamoya disease. HC, healthy controls. M, male; F, female. L, left; R, right. Duration of symptoms indicates the duration from the first symptom until the hospitalization.
